# Local Chemical Enhancement and Gating of Organic Coordinated Ionic‐Electronic Transport

**DOI:** 10.1002/adma.202406281

**Published:** 2024-11-19

**Authors:** Tamanna Khan, Terry McAfee, Thomas J. Ferron, Awwad Alotaibi, Brian A. Collins

**Affiliations:** ^1^ Department of Materials Engineering Washington State University Pullman WA 99164 USA; ^2^ Department of Physics Washington State University Pullman WA 99164 USA; ^3^ Lawrence Berkeley National Laboratory Berkeley CA 94720 USA

**Keywords:** chemical sensing, interfacial transport, ion mobility and conductivity, organic electronics, organic mixed ionic‐electronic conductors (OMIEC)

## Abstract

Superior properties in organic mixed ionic‐electronic conductors (OMIECs) over inorganic counterparts have inspired intense interest in biosensing, soft‐robotics, neuromorphic computing, and smart medicine. However, slow ion transport relative to charge transport in these materials is a limiting factor. Here, it is demonstrated that hydrophilic molecules local to an interfacial OMIEC nanochannel can accelerate ion transport with ion mobilities surpassing electrophoretic transport by more than an order of magnitude. Furthermore, ion access to this interfacial channel can be gated through local surface energy. This mechanism is applied in a novel sensing device, which electronically detects and characterizes chemical reaction dynamics local to the buried channel. The ability to enhance ion transport at the nanoscale in OMIECs as well as govern ion transport through local chemical signaling enables new functionalities for printable, stretchable, and biocompatible mixed conduction devices.

## Graphical abstract

Record ion mobility and conductivities are revealed within a nanoscopic interfacial superhighway of an organic mixed ionic‐electronic conductor. Fast ion transport can be controlled by hydrophobicity of molecules local to this channel, effectively gating ion access to the superhighway. This mechanism is used in a novel chemical sensing device which detects the dynamics of a local, buried chemical reaction.

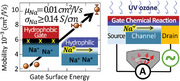

## 1 Introduction

Among advantages of organic mixed ionic‐electronic conductors (OMIECs) over inorganic materials is their high volumetric capacitance for ions to inject and swell the material^[^ ^1^ ^]^ as well as a lack of dangling bonds at surfaces that interfere with ion transport or other properties.^[^ ^2^ ^]^ In particular, OMIEC devices have been shown to outperform their inorganic counterparts in the transduction of electrophysiological signals in the body and brain.^[^ ^3^ ^]^ The signaling dynamics, however, are limited by the slower ion motion relative to charge transport. Increasing ion mobilities and conductivities would have a large impact on device response time and bandwidth for sensing and computing applications.^[^ ^4^ ^]^ Nanoscale control of ion motion is critical as well for these applications. In biological processes, ion transport is often manipulated at the nanoscale via molecules of opposing surface energies where, for example, neurons actuate signaling using hydrophilic molecules to attract ions across a hydrophobic cell membrane.^[^ ^5^ ^]^ This raises the possibility of controlling and accelerating ion motion through designed nanomorphology and local hydrophilicity.

## 2 Results and Discussion

**Figure 1:**
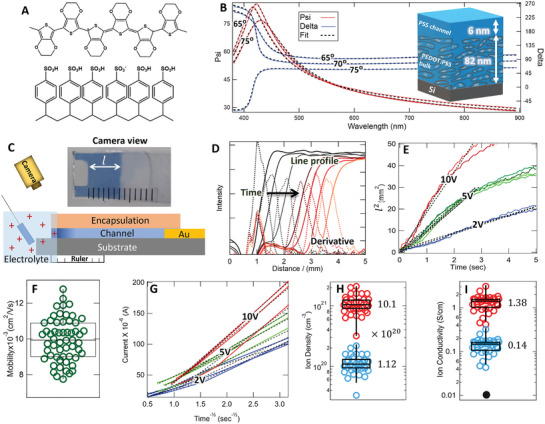
Establishing an Ion Superhighway A) Chemical structure of the PEDOT:PSS. B) Two‐parameter VASE analysis of a PEDOT:PSS thin film to characterize the bilayer. Inset shows the measured thicknesses. Three other film thicknesses are consistent with this as shown in Section S2 (Supporting Information) C) Schematic of ECMF experiment setup. The camera view shows the image of a partially dedoped (blue) device. D) Gaussian smoothed CCD red‐channel intensity profiles (solid lines) and corresponding derivatives (dashed lines) acquired from video frames locate the dedoping front *l*. E) Dedoping transients for multiple trials on a single mixed conduction device at several driving voltages. Solid and broken lines represent the data and the linear fit, respectively (details in Section S4, Supporting Information). F) Ion mobility statistics from different devices, trials, and driving voltages with a PVA encapsulation layer. (*n* = 49) (G) Current versus $\frac{1}{\sqrt{t}}$ and linear fits for a PVA mixed conduction device (analysis details in Section S5, Supporting Information). The time ranges used for the fits are identical to those used in E). H) Ion density, *p*, and I) ion conductivity, σ_*ion*_, statistics from bounding scenarios of ion distribution in the hydrated channel. Explicit posted values are from the average for each scenario (*n* = 31).

We accomplish this in a model polyelectrolyte OMIEC Poly(3,4‐ethylenedioxythiophene)‐poly(styrene sulfonate), (PEDOT:PSS, structure **Figure** **1**A) where hydrophobic, hole‐conducting PEDOT oligomers associate with PSS chains, which are electrically insulating and hydrophilic. These complexes form PEDOT‐rich gel nanoparticles within a PSS‐rich matrix.^[^ ^6^ ^]^ Our previous work has indicated heterogeneous ion mobility around these nanoparticles with the hydrophobic PEDOT repelling and disrupting ion transport.^[^ ^7^ ^]^ Here we demonstrate that an interfacial PSS nanolayer in mixed conduction devices can centralize and accelerate ion transport, and that this channel can be chemically gated by the surface energy of local molecules. Previous studies on phase separation^[^ ^8^, ^9^ ^]^ and surface analyses^[^ ^10^, ^11^ ^]^ indicate a spontaneous PSS wetting layer due to differential surface energies of the components. Water contact angle (WCA) measurements show the PEDOT:PSS surface – initially equivalent to a pure PSS layer – becomes more hydrophobic with a water rinse just before PSS crosslinking (See Experimental Section and Section S1, Supporting Information). The change is consistent with the removal of a hydrophilic PSS top layer, exposing the hydrophobic PEDOT‐rich bulk beneath.

The geometry of the PSS interfacial layer is characterized by variable angle spectroscopic ellipsometry (VASE). Figure 1B displays the two‐parameter VASE analysis of a PEDOT:PSS bilayer using optical models separately developed for a PEDOT‐rich bulk and a pure PSS interfacial layer (Section S2 and Table S2, Supporting Information). This analysis on multiple films of different thicknesses gives consistent results of 6–8 nm of PSS layer atop a PEDOT‐rich bulk (Table S1, Supporting Information) and is consistent with a high surface energy wetting layer. Other potential layer combinations (including no wetting layer) result in comparatively poor fits. The optical model of the PEDOT‐rich bulk independently agrees with transmission UV–vis absorbance spectroscopy measurements with no adjustable parameters (Section S3, Supporting Information), further supporting the accuracy of the analysis here. This measurement also agrees with previous X‐ray reflectometry measurements.^[^ ^12^, ^13^ ^]^

Ion mobility of the channel is measured in mixed conduction devices using an electrochromic moving front (ECMF) experiment.^[^ ^14^, ^15^ ^]^ The geometry and mechanism of the experiment are shown in Figure 1C with an example video frame of a partially developed device. In the device, ions enter from the electrolyte to dedope PSS^−^ from the PEDOT^+^, converting it to the neutral PEDOT^0^ with the conduction of a hole to the Au counter electrode. This changes PEDOT's color from transparent to blue.^[^ ^16^, ^17^ ^]^ Being the slow carrier, ions control the reaction rate, and the electrochromic front reveals the ion mobility under the series resistor model developed by Stavrinidou and coworkers^[^ ^12^, ^13^ ^]^ (Figure 1D,E, details in Section S4, Supporting Information), where the limited ion mobility in the developed (blue) segment of the device controls the overall current relative to the higher mobility electrons in the doped segment.

To attract ions into the PSS‐rich top channel, we use a hydrophilic poly(vinyl acetate) (PVA) encapsulation layer directly in contact with the PSS channel. Figure 1F represents the statistics of measured mobility values over multiple devices, trials, and driving voltages with $\mu_{Na}=10.0\left(0.2\right)\times 10^{-3}\frac{cm^{2}}{Vs}$. This is an order of magnitude higher than previous reports of similar films^[^ ^7^, ^12^ ^]^ and is consistent with ions diffusively collecting into the top PSS interfacial layer due to the high local surface energy of PVA. Lacking PEDOT gel particles, ions can move more freely in this interfacial channel than in the bulk, representing an ion superhighway. The combined effect of the interfacial superhighway and bulk layers beneath act as parallel ion resistors within the dedoped segment of the device, and the measured effective mobility is a weighted sum of the ion mobilities in both layers (see Section S4, Supporting Information for more details). This effective mobility we measure is dominated by the superhighway if the mobility is significantly higher than the bulk which is true in this case (vide infra). Supporting this view, we see the moving front velocity decrease as the channel thickness is increased (Figure S9, Supporting Information), due to the interfacial nature of the superhighway that bottlenecks ions from dedoping the increasing volume of PEDOT in the bulk. Notably, the mobility we measure here is more than an order of magnitude higher than the electrophoretic mobility of Na^+^ in water $\mu_{Na}^{EP}=0.52\times 10^{-3}\frac{cm^{2}}{Vs}$ ^[^ ^12^, ^18^ ^]^ and to our knowledge represents the highest ion mobility reported for any ion in any material.

The DC ion conductivity, σ_*ion*_ =  *ep*µ (elementary charge *e*), is calculated from mobility and ion density, *p* measured via current transients acquired simultaneous to the ECMF experiment (Figure 1G details in Section S5, Supporting Information). We calculate the bounding values of *p* in Figure 1H via two limiting possibilities of the vertical ion distribution in the hydrated channel: Homogeneous density throughout the entire film (low bound) or concentrated transport only in the PSS interfacial channel (high bound). The lower bound is consistent with previous measurements on bulk PEDOT:PSS films,^[^ ^13^ ^]^ while the upper bound approaches the estimated anion density of swollen PSS (Section S10, Supporting Information). The corresponding σ_*Na*_ statistics and range (Figure 1I) result in values between 14 and 140 times higher than previous reports at similar hydration levels^[^ ^19^ ^]^ (black dot in Figure 1I). This range is equivalent to *proton* conductivity σ_*H*_ in state‐of‐the art fuel cell membranes such as Nafion 117 and other recent advanced polymer membranes including covalent organic frameworks.^[^ ^20^, ^21^ ^]^ The high end approaches enhanced σ_*H*_ accomplished through nanoscale alignment and confinement effects of water channels^[^ ^22^ ^]^ and solid state superionic conductors.^[^ ^23^ ^]^

**Figure 2:**
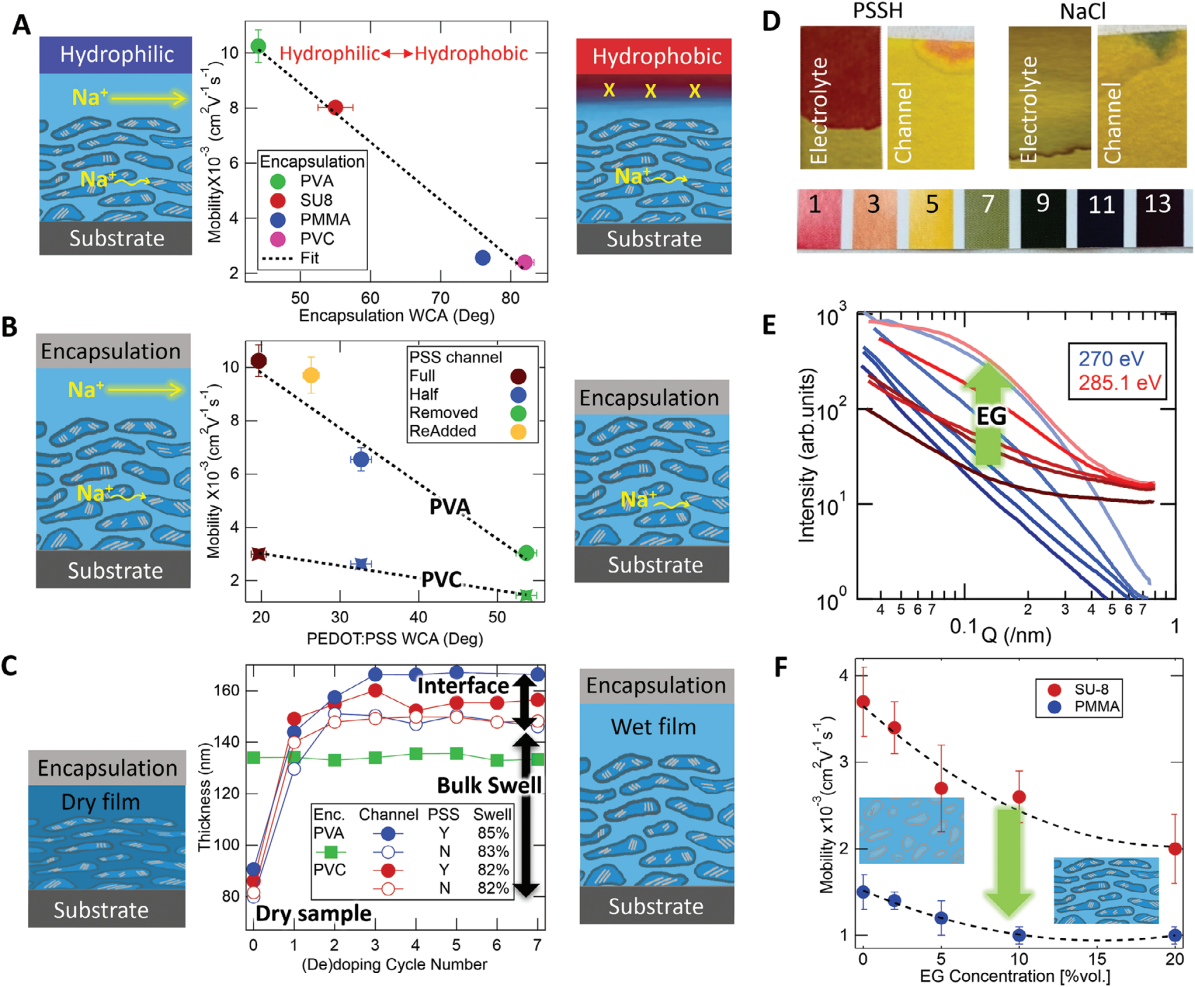
Control Mechanisms of Ion Transport A) Ion mobility versus WCA of four different encapsulation layers. Line as a guide to the eye. SU‐8 is a negative photoresist; PMMA is polymethyl methacrylate; and PVC is poly(vinyl chloride). B) Na^+^ Ion mobility versus WCA of the PEDOT:PSS channel surface, which varies due to water sonication of the channel that increasingly removes the PSS‐rich top layer. Lines are guides to the eye. Devices with no PSS removal are designated as “Full”, PSS removal after PSS crosslinking “Half”, PSS removal before crosslinking “Removed”, and “ReAdded” means pure PSS is spin‐coated on top of a “Removed” layer (see “PEDOT:PSS Films” in Experimental Section for details). Devices with two different encapsulation layers are shown: hydrophilic (PVA) and hydrophobic (PVC). C) VASE analysis of channel swelling for the devices in Figure 2B as a function of ECMF dedoping cycle. Cycle 0 is the thickness in the initial dry state. Solid symbols are devices with the “Full” PSS channel while open symbols are for devices with the PSS channel “Removed”. Green squares are the PVA encapsulation layer thickness. D) pH litmus test on dedopoed devices without encapsulation using PSSH and NaCl electrolyte. E) C‐edge RSoXS profiles of PEDOT:PSS films at 270 eV (blue) and 285.1 eV (red, PEDOT carbon 1s → π* transition). The dark to light color indicates increasing in EG content of 0, 2, 5, 10, and 20 vol%. F) Ion mobility of PEDOT:PSS films with SU8 (red) and PMMA (blue) encapsulating layers versus EG cosolvent concentration. Note: PSS was “Half” removed for this series [e.g., blue symbols in (B)], reducing the mobility and interfacial effects compared to those in (A), similar to the processing of devices in previous reports using SU‐8 encapsulation.^[^ ^7^, ^12^ ^]^ Insets depict enhanced gelation from EG. All mobility statistics from multiple trials and driving voltages on at least three identically prepared devices.

The concentration of fast ion transport occurring in such a narrow region of the channel provides an opportunity to control this transport locally. To accomplish this, we use channel encapsulation layers of increasing surface energy as documented via solubility analysis (Section S6 and Table S3, Supporting Information) and measured via WCA (Figure S1F–I, Supporting Information). **Figure** **2**A shows a plot of Na^+^ ion mobility versus encapsulation WCA for several different encapsulation layer polymers. The plot reveals a dramatic reduction of Na^+^ mobility as local hydrophobicity from the encapsulating layers increases, eventually returning µ_*Na*_ to values reported in the literature.^[^ ^7^ ^]^ This further supports higher ion mobility originating from the free motion of ions in the PSS interfacial channel rather than the bulk. A local hydrophilic layer attracts water and Na^+^ from the electrolyte (presumably from traditional diffusion) to transport through the top PSS channel whereas a hydrophobic material repels water from the channel, driving the ions out. This effectively gates access to this interfacial channel via the local encapsulant's surface energy (see schematics in Figure 2A). Such record mobilities and a gating effect are additionally seen for proton and potassium ions in H‐PSS and KCl electrolytes (Figure S11, Supporting Information).

In Figure 2B, we further investigate the gating mechanism by increasingly removing the PSS interfacial layer via water sonication before and after PSS crosslinking to produce a “Full” PSS interfacial layer, a “Half” PSS layer, and a “Removed” PSS layer. (see “PEDOT:PSS Films” in Experimental Section). PSS removal is confirmed via VASE analysis showing a reduction in thickness from this treatment and the top surface exhibiting a higher WCA (Figure S1A–C, Supporting Information). Devices were fabricated from these films using the most hydrophobic (PVC) encapsulation as well as the most hydrophilic (PVA) encapsulant. Their Na^+^ mobilities are presented versus WCA of the PEDOT:PSS channel surface in Figure 2B, revealing little change of ion mobility for the hydrophobic device compared with a dramatic reduction in mobility for the hydrophilic device as the PSS top layer is increasingly removed. In particular, the ion mobility in the hydrophilic device with PSS “Removed” becomes similar to the lower mobility of the hydrophobic device. The lower mobility in the hydrophobic device is consistent with previous reports of mobility for bulk PEDOT:PSS transport^[^ ^7^ ^]^ and is consistent with ions being repelled out of the ion superhighway by the local hydrophobic layer. The result of low mobility in the PVA device establishes that the fast ion transport does not occur in the hydrophilic PVA encapsulation layer itself. Intriguingly, when a PSS layer is recast after the “Removed” treatment, the record high Na^+^ mobility is re‐established (Figure 2B, “ReAdded” yellow point). The dramatic reduction in mobility upon PSS removal and the return of record mobility when the PSS layer is replaced provides further evidence that the high‐mobility channel is in the PSS‐top layer only. The fact that high mobility, furthermore, only occurs with the PVA device reveals that high mobility will only occur if both conditions are satisfied: The presence of a PSS top layer and a high surface energy encapsulant.

Further evidence of the high mobility originating in the PSS interfacial layer is revealed by VASE swelling measurements on the “Full” and PSS “Removed” hydrophilic and hydrophobic devices described above (example analyses in Section S9, Supporting Information). The total channel thickness (combined PSS‐top layer and PEDOT‐rich bulk) is plotted in Figure 2C after each ECMF dedoping cycle. The “zeroth” cycle thickness of the channel's dry state is used to calculate the final channel swelling, quantified in the Figure 2C inset table (calculation details in Table S5, Supporting Information), revealing ≈83% channel swelling for all devices. Also, for all devices, a steady‐state swollen thickness is not achieved until after the third dedoping cycle. Similarly, a steady‐state mobility value is not measured until this cycle. This indicates that full hydration is required for the fastest transport. Only steady‐state mobilities were used in our evaluation of mobility. For both hydrophobic and hydrophilic devices, channels with PSS (solid circles) have systematically higher thicknesses than without the PSS (open circles) enabling us to measure swelling of just the PSS top layer alone. The swelling of the PSS interfacial channel in the hydrophobic PVC device is equal to the bulk swelling (83%). However, PSS top layer swelling in the hydrophilic PVA device is significantly higher at 102%, demonstrating that the hydrophilic PVA layer actively attracts electrolytes into the PSS interfacial channel.

Measuring Na^+^ effective mobility above that for electrophoretic transport is remarkable, since polymer chain hopping is typically a slower process than vehicle transport in a solvent.^[^ ^12^, ^19^ ^]^ Delamination of the encapsulation layer from the channel might allow for swift water transport that is accelerated due to confinement. However, delamination is easily detected by a lack of moving front in the ECMF experiment (Figure S12, Supporting Information) and is not included in calculating the mobility. The VASE thickness analysis further rules out delamination with the maximum measured swelling that agrees with previously reported values for polymer‐mediated transport,^[^ ^12^ ^]^ which indicates a similar mechanism is likely responsible here. The VASE analysis also reveals a constant PVA layer thickness regardless of channel hydration (Figure 2C green trace), providing further evidence that ion transport occurs only in the PEDOT:PSS channel and does not penetrate the PVA layer. On the other hand, litmus experiments (Figure 2D) confirm the transport of Na^+^ rather than potentially faster protons in the channel, while using NaCl as an electrolyte.

A full analysis of the electrokinetic mechanism responsible for this record ion mobility is outside the scope of this work. However, the evidence accumulated here is consistent with confinement effects of the nanoscale channel similar to that previously reported for fast proton transport in Nafion nanofibers.^[^ ^22^ ^]^ Such enhanced transport can occur when electric double layers at opposite surfaces of a nanochannel overlap for example when the Deybe length is the same or larger than the width of the channel itself.^[^ ^24^, ^25^ ^]^ Super‐ionicity^[^ ^23^ ^]^ is not ruled out here either. Grotthuss‐like transport that involves Na^+^ rather than protons is also a potential mechanism, where instead of individual ion transport through the entire distance, injected ions merely displace another at a nearby site, causing the nearby ion to jump and so on. Also conceivably contributing to the high ion mobility is the ease of ions traveling a linear path along the electric field rather than tortuous paths around the PEDOT gel particles within the bulk of the layer.

We further explore the interplay between the superhighway effect and internal nano‐morphology of the channel by varying the ethylene glycol (EG) cosolvent content during casting the PEDOT:PSS layer, which was previously shown to vary the heterogeneity between PEDOT‐rich gel particles and the PSS‐rich matrix.^[^ ^7^ ^]^ Figure 2E displays resonant soft X‐ray scattering (RSoXS) profiles at 270 eV (blue, non‐resonant) and 285.1 eV (red, PEDOT resonance) of the five‐sample series cast from increasing EG content. The profiles reveal a scattering feature centered at *Q* ≈ 0.12 *nm* ^−1^ ($\frac{2\pi }{Q}\approx 52nm$). Scattering enhancement at resonance indicates that these features come from the chemical heterogeneity between the PEDOT‐rich gel nanoparticles and the PSS matrix, which can be determined by considering the scattering contrast functions published earlier.^[^ ^7^ ^]^ The feature's scattering intensity increases dramatically with EG content, indicating more aggregation of PEDOT‐rich domains in the film which promotes electron transport but impedes ion transport.^[^ ^7^, ^9^, ^26^, ^27^ ^]^ Reduction of ion mobility measured here and shown in Figure 2F (67% and 54% for PMMA and SU8 coated devices, respectively) agrees with our earlier reported 61% reduction.^[^ ^7^ ^]^ Despite this similarity, ion mobility between the mildly hydrophilic (SU8) encapsulation layer is consistently >2x higher than devices with the hydrophobic (PMMA) layer, indicating that, the effect of the interfacial PSS top layer is separate from the effect of nanodomain morphology and affects ion transport more significantly.

**Figure 3:**
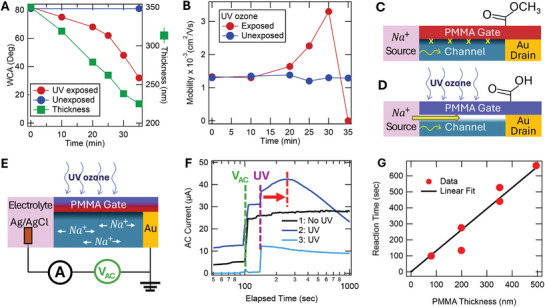
Sensing a Local Chemical Reaction A) Top surface WCA and thickness (VASE) of a PMMA encapsulation layer with accumulated UV ozone exposure time. B) Ion mobility (ECMF) as a function of accumulated UV exposure time in a PMMA‐coated device exposed to UV (red) and unexposed (blue). Figure S15 (Supporting Information) shows the experiment reproduced twice more. Schematic of “transistor” devices C) before and D) after UV ozone exposure. UV ozone reacts with the PMMA encapsulation layer to turn the PMMA ─OCH_3_ group to ─OH, acting as a chemical gate signal. E) Schematic of device setup for electrical detection of a chemical change. An AC voltage (*V*_AC_) oscillates ions in place (100 Hz) with the AC current sensing ion mobility. F) AC current transients for three sequential runs on one device. The green and violet lines indicate when V_AC_ was turned on and the UV exposure was activated, respectively. The peak in current (red line) represents maximum ion mobility when the chemical reaction reaches the buried channel with the red arrow representing the chemical reaction time. G) Chemical reaction time as a function of PMMA thickness (linear fit) under UV exposure.

Having established that fast ion transport is localized to the PSS interfacial layer and can be controlled via the local encapsulant surface energy, we set out to use this new mechanism in a device capable of sensing a buried chemical reaction. We choose to sense an oxidation reaction of PMMA at the buried channel. Exposure of PMMA to UV in the presence of oxygen causes a photooxidative reaction of the methacrylate groups, forming peroxy radicals that diffuse into the layer and undergo secondary reactions to hydroxyl and carbonyl groups.^[^ ^28^, ^29^ ^]^ This transitions the polymer from hydrophobic to hydrophilic, causing the PMMA encapsulant to act as a chemical gate of the PSS interfacial layer. We exposed PMMA‐encapsulated devices to UV ozone to transform the encapsulation “gate” chemically. **Figure** **3**A shows that over the course of accumulated UV exposure, the PMMA top surface indeed progressively becomes more hydrophilic (WCA from 82° to 32°), and the layer itself becomes thinner (350 to 220 nm) due to the reaction. After 20 mins exposure, a dramatic 130% increase in mobility is detected (Figure 3B), which maximizes at 30 mins. Upon further exposure (35 mins), ion transport halts entirely. In Section S11 (Supporting Information) this behavior is reproduced several times on separate devices.

These events are depicted in Figure 3C,D, where the initially hydrophobic PMMA gate blocks the top interfacial channel, keeping the ion mobility low (Figure 3C). Upon accumulated UV exposure, the oxidation reaction in the PMMA propagates to the bottom of the encapsulation layer, causing an increased local surface energy in the interfacial PSS channel (Figure 3D). This attracts ions into the ion superhighway, actively switching the device into the high conductivity “on” state. The chemical switching from ion repulsion to attraction in the interfacial channel is identical to the function of the accumulation layer in a thin film transistor.

We can additionally detect this local chemical reaction in situ via electrical sensing rather than relying on the ECMF experiment. The process is shown in Figure 3E where an AC voltage oscillates the ions within the channel while the AC current senses the ion mobility (details in Section S12, Supporting Information). Figure 3F plots the AC current transients for three trials: A control trial (no UV) and then two sequential trials with UV exposure. The control trial demonstrates the stability of the AC current over time while the device is held at an open circuit DC bias (*V_oc_*). In applying UV (violet vertical line) there is an initial photocurrent step and subsequently a gradual peak in AC current, indicated by the red vertical line. A second UV trial has no current peak. This sequence is consistent with the irreversible chemical reaction burrowing through the PMMA layer, activating the ion superhighway via the hydrophilic product. The UV photocurrent step indicates immediate UV penetration and starts the clock of the chemical reaction. The separation in time between this UV step and the AC peak is the reaction time (indicated by the arrow) for the peroxy radicals to traverse the encapsulation layer. This suggests separate detection between light exposure and the PMMA chemistry, and therefore, an electrical detection of a buried chemical reaction.

If the peak in the AC current after the UV step represents the time for the reaction front to arrive at the buried channel, then thinner PMMA layers should activate the channel earlier under a constant reaction rate. This is confirmed in Figure 3G, where the reaction time (time to the peak in current transients shown in Figure S17, Supporting Information) is found to be linearly proportional to the PMMA layer thickness with a reaction rate of 7.6(4) Å s^−1^. This demonstrates the electrical detection of a chemical reaction moving front within a solid‐state device. Such a capability to both sense a local chemical reaction at a buried interface and to quantify reaction rates through a thin film could be highly useful in thin film devices or chemical sensors.

## 3 Conclusions

Overall, the mode of control demonstrated here over ion transport opens new opportunities in device functionality and biomimicry. For example, we control the presence of ions in fast channels through local surface energy, which is the same mechanism utilized in cell walls to guide ions through channels.^[^ ^5^ ^]^ The sensing mechanism could go beyond characterizing a local reaction. For example, it could be further employed in neuromorphic paradigms that use local chemical environments to gate ion pathways and even change those environments through (reversible) reactions catalyzed by the ions themselves. Given that ion transport is the slow carrier in mixed conduction, accelerated ion transport demonstrated here enables increased electrochromic switching speeds,^[^ ^30^ ^]^ electrochemical transistor and actuator bandwidths in soft robotics,^[^ ^31^, ^32^ ^]^ and write/read speeds in neuromorphics.^[^ ^33^, ^34^ ^]^ However, our results reveal the difficulty in measuring the intrinsic mobility of ions in a multi‐component material such as a polyelectrolyte OMIECs, evidenced by an order of magnitude difference in mobility depending on access to the ion superhighway. The measured effective mobility above electrophoretic mobility additionally indicates new potential mechanisms of ion transport that push the limits of what is possible with ion transport. The materials used for both the gate and channel are by no means extreme in their surface energies, and this effect is likely to increase as those limits are approached. Thus, combining this control with new materials is likely to result in new horizons for OMIEC‐based devices.

## 4 Experimental Section

### PEDOT:PSS Solution Preparation

PEDOT:PSS was purchased from Heraeus (Clevios 1000) and mixed with 1 vol.% of the surfactant Dodecylbenzene Sulfonic Acid (DBSA) (diluted with DI‐water in the ratio of 1:3), 0–20 vol.%. cosolvent EG, and eventually PSS crosslinker 0.1 vol.% Glycidoxypropyltrimethoxysilane (GOPS) – all are purchased from Sigma Aldrich. The solution is sonicated twice for 15 mins before and after stirring the solution for around 4 h. For RSoXS nanomorphology characterization only 10 mins of the sonication is performed to disperse the gel particles. After stirring/sonication, the solution is filtered with a 1.2 µm cellulose acetate filter. The GOPS crosslinking agent is added to the solution just before (2–3 mins) the deposition of the thin film.

### PEDOT:PSS Films

The solution was spincast on the substrates at 2000 rpm for 1 min and soft baked (90 °C for 1 min) to remove water, resulting in a ≈50 nm film (measured by VASE). Typically, two spin‐coating cycles were done before a final hard bake (140°*C* for 40 mins in a vacuum oven) which activated the GOPS to crosslink the PSS chains in the film, immobilizing them. Due to the bottlenecking effect of transport in the interfacial layer that slowed the apparent velocity of ions dedoping the PEDOT bulk, thinner films (80 nm by VASE) were used appreciably than previous reports (400 nm).^[^ ^12^, ^17^ ^]^ The resulting films were optionally sonicated in DI‐water for 1 min before (PSS layer “Removed”) or after (PSS layer “Half” removed) the hard bake for the experiment in Figure 2B. In addition to those samples in Figure 2B, the “Half” removed protocol was additionally applied to all films in Figure 2F and is the procedure used in previous publications.^[^ ^12^, ^17^ ^]^

### Mixed Conduction Devices

A glass substrate of 1.5″ × 0.75″ was cleaned by sonicating 20 mins in detergent, 10 mins in DI‐water, 20 mins in acetone, and 20 mins in Isopropyl Alcohol (IPA) followed by blow drying with dry Nitrogen. After 20 mins of UV ozone cleaning, 40 nm of Au was deposited (≈0.25″) on one side of the glass substrate (Figure 1C) using a physical vapor deposition (PVD, base pressure ≈10^−7^ Torr, 0.04 nm s^−1^). Next the PEDOT:PSS film was deposited as described above. PVA, PVC, and PMMA (purchased from Polymer Source) are then dissolved 40 g L^−1^ in Chloroform, Tetrahydrofuran or Cyclohexane, and Toluene, respectively. VASE analysis on post‐treated layers helped to select orthogonal solvents for the encapsulant materials that did not affect the bilayer channel beneath (see details in Section S13, Supporting Information). They were spincast atop the PEDOT:PSS film to create a ≈130–300 nm encapsulation layer (measured via VASE). Next, the organic film was removed from the gold electrode to establish a connection with the electrode while dedoping via dipping in Acetone. The end of the channel opposite the electrode was dipped into the IPA to remove the encapsulation material from the film which will allow the access of the electrolyte solution to the channel. Afterward, the device was baked at 120 °*C* for 30 mins to eliminate residual solvents. On the other hand, SU8 was spin cast and patterned with UV exposure followed by baking the device. A pocket hole was created during photolithography and both SU8 and film were removed from there using a DI‐watered cotton swab.

### Electrochromic Moving Front (ECMF) Experiment

The mixed conduction experiment and theory was described in detail in previous publications.^[^ ^17^, ^18^ ^]^ In this work, the devices and an Ag/AgCl electrode of 2 mm × 4 mm (purchased from A‐M Systems) are submerged into 100 mm of either NaCl, KCl, or HPSS electrolyte. A bias of 2–10 V drive dedoping the film, while a Logitech webcam records video of the process. A ruler fragment taped underneath the device calibrates distance. Dedoping was reversed (redoping) by short‐circuiting the device electrodes. No reverse bias was ever applied to the devices, and thus there was no driving force for anion injection, only cation. 10–12 dedoping/redoping cycles were conducted to investigate ion motion, each creating a dedoping transient and each with a distributed pattern of driving voltages. The process of changing the voltages for doping and redoping cycles was computer automated where each cycle occurs over 150 sec: dedoping for 30 sec, redoping for 2 mins (see Figure S10, Supporting Information). The first two cycles achieved a steady state hydration of the PEDOT:PSS channel^[^ ^33^ ^]^ with the rest analyzed to determine statistics of the ion mobility. For each preparation condition, at least three devices were tested to increase statistics and reduce errors. Dewetting of the encapsulation layer from the channel was clear from the dramatically different dedoping transients and removed from the mobility statistics (See Figure S12, Supporting Information). In each cycle, an initial linear transient gave way to a plateau where ion motion halts and the channel hydration ends. Only the linear portion of each transient was analyzed (Figure 1e).

### Ion Conductivity Measurements

Simultaneous to the optical ECMF experiment, electrical current through the channel was recorded (2450 Keithley SMU) to calculate the ion density, *p* and ion conductivity, σ. The exact same time ranges used to calculate µ_*Na*_ were considered to determine *p* such that both used simultaneous measurements. See Section S5 (Supporting Information) for experimental setup and further details. The cross‐sectional area required to calculate *p* was derived from channel thickness measurements (VASE) on the fully hydrated films.

### Variable Angle Spectroscopic Ellipsometry (VASE)

A Woollam Alpha‐SE was used to characterize the bilayer morphology of the PEDOT:PSS channel. Films were cast on 1.5″ x 1.5″ bare Si wafers but otherwise identical to mixed conduction devices (same cleaning procedures). Optical models for the PSS‐rich top layer and PEDOT‐rich bulk were created from VASE measurements on separate samples and fit without variation to the bilayer film using only two parameters: the thickness of the top and bulk layers.

For the swelling experiment, optical models for PVA and PVC encapsulation layer were made by spin‐casting the solvents on Si substrates and fitting the VASE data to an isotropic B‐Spline model. Mixed conduction devices (described earlier) were made and fitted using the PVA or PVC optical constants for the encapsulation layer and a biaxial model for the channel to measure the thickness of the swollen PEDOT:PSS channel. For further details see Section S9 (Supporting Information) and tables therein. All analysis was completed in the Woollam Complete Ease analysis software.

### Resonant Soft X‐ray Scattering (RSoXS) Samples and Experiment

SiN windows with nitride thickness 100 nm (Norcada) were used for soft X‐ray characterization followed by spin casting of PEDOT:PSS as described earlier after cleaning the substrate in the UV ozone for 20 mins. Note that both the devices and films for scattering involved a water rinse after a hard bake, representing a partial removal of the PSS channel (same process as blue symbols in Figure 2B). Also the PEDOT:PSS thickness was double that used in typical devices to enhance the RSoXS signal. This had the effect of reducing the effective mobility in the series. Scattering was performed at beamline 11.0.1.2 of the Advanced Light Source, Berkeley National Laboratory using the instrument and standard procedures described elsewhere.^[^ ^34^ ^]^ All data shown were taken using horizontal linear polarization and a CCD exposure time of 150 sec. The films were not coated with any encapsulated material for X‐ray characterization.

### Activating the Channel with a Chemical Reaction

PMMA‐based mixed conduction devices were used. The films were sonicated for 1 min before the hard bake. An identically cast PMMA film on a bare Si wafer enabled thickness monitoring (measured by VASE) as a function of UV ozone exposure. A UV ozone cleaner (HELIOS‐500) capable of 184.9 and 253.7 nm ultraviolet radiation with a mercury lamp (intensity 19.4 mW cm^−2^) was used. Between each UV–ozone exposure, the encapsulation layer WCA was measured immediately before and after the ECMF (mobility) experiment along with the thickness and WCA of the PMMA/Si double. The ion mobility from two devices, exposed and unexposed, was measured simultaneously. The entire experiment was completed three times (separate sets of devices) to assure reproducibility (see Figure S15, Supporting Information for repeat trials).

### In Situ Electronic Detection of Channel Activation

A mixed conduction device with PMMA encapsulation was used. While dedoping the device using the ECMF experiment both AC and DC currents and voltages were measured. See Figure S16 (Supporting Information) for the experimental setup. The electrical signals were measured from both unexposed devices and devices exposed to UV ozone. The unexposed device was used as a control. The ECMF measurements were acquired at 5 V driving voltages.

The device was first dedoped/redoped for a few cycles until the channel reaches to the complete hydration. To initially inject ions a DC voltage was first applied until the dedoping front gets half the distance along the channel. Then the voltage was decreased to the open circuit voltage *V_oc_* to nullify the DC current. By maintaining *V_oc_* in the device, injected ions were kept in the channel. During this time, an AC amplitude was applied to the device. The AC current was then proportional to the device ion mobility under the series resistance model described in Section S12 (Supporting Information).

### Statistical Analysis

1) Pre‐processing of ECMF data involved extracting a line scan of the red channel video images along the PEDOT:PSS channel. These line scans were gaussian smoothed and differentiated to locate the precise moving front position at each frame in the video. 2) All final values for mobility and conductivity were the mean as the best estimate and the standard error (standard deviation divided by the square root of n measurements). 3) Sample size (*n*) for each statistical analysis is stated in the Figure caption for that analysis. 4) No statistical methods were employed in this study to assess significant differences. 5) Igor Pro was used for all statistical analyses.

## Conflict of Interest

The authors declare no conflict of interest.

## Author Contributions

BAC and TK designed the experiments, conducted data analysis, and drafted the manuscript. TM helped design and develop ECMF experiments. TK prepared the samples and carried out ECMF experiments, VASE, and UV vis measurements. TF performed RSoXS measurements and TK analyzed the data. AA automated the ECMF experiment for ion density and conductivity measurements. All authors contributed significant effort to this project.

## Supporting information



Supporting Information

## Data Availability

The data that support the findings of this study are available from the corresponding author upon reasonable request.
