# Supplementary material for: Local Chemical Enhancement and Gating of Organic Coordinated Ionic‐Electronic Transport
Source: Adv Mater. 2024 Nov 19;37(5):2406281. doi: 10.1002/adma.202406281 (PMC11795723; doi:10.1002/adma.202406281)
Supplement: Supplementary file 1 — Supporting Information [file ADMA-37-2406281-s001.docx]

Local Chemical Enhancement and Gating of Organic Ionic-Electronic Transport

Tamanna Khan^1^, Terry McAfee^2,3*^, Thomas Ferron^2*^, Awwad Alotaibi^1*^, Brian A. Collins^1,2*^

^1^ Department of Materials Engineering, Washington State University; Pullman, WA, USA

^2^ Department of Physics, Washington State University; Pullman, WA, USA

^3^ Lawrence Berkeley National Laboratory, Berkeley, CA, USA

**Supplementary information**

# Water Contact Angle (WCA) Measurements

Contact angles are measured using a drop of water on the target surfaces. Then an image is acquired from the side with a camera just after the drop has stopped dispersing on the substrate’s surface and angle is measured with the help of an image protractor. For the UV ozone exposure experiment, the WCA is measured each time after exposure.


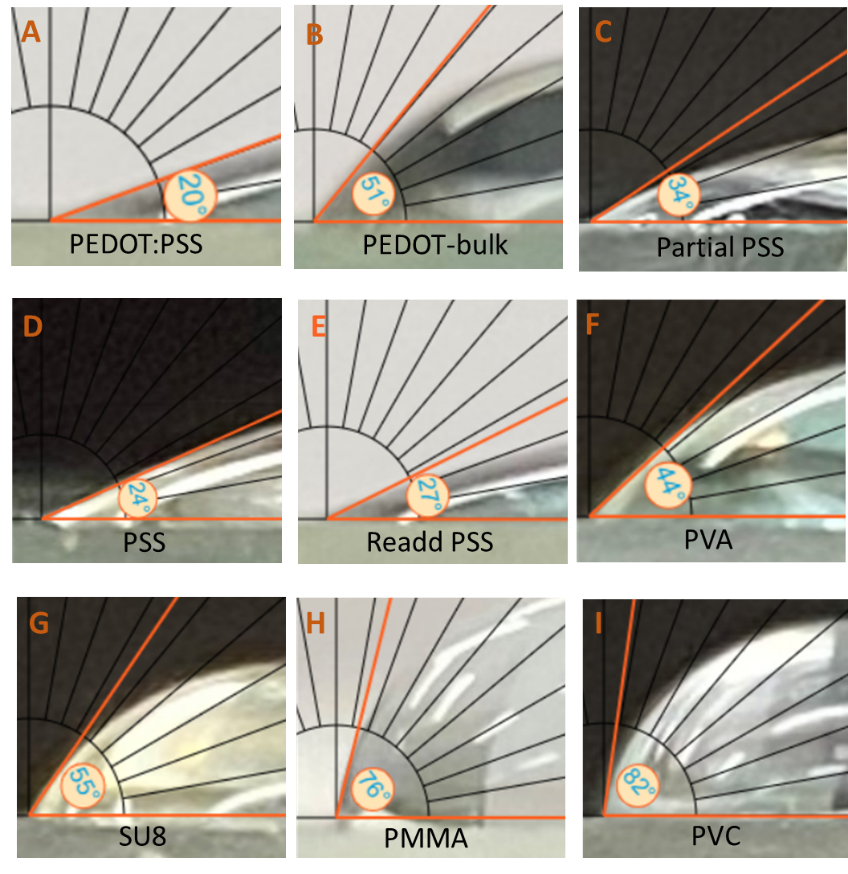


Figure S1: Water Contact Angles (WCA) Measurements. WCA with a drop of DI-water on the surface of PEDOT:PSS film when (A) PSS is not removed (unsonicated), (B) PSS is fully removed (sonicated before film crosslinking), (C) PSS is partially removed, (D) PSS with GOPS and (E) PSS is re-added after removing it. A, C, and E show similar results are expected. WCA on (F) PVA, (G) SU8, (H) PMMA, and (I) PVC.

# VASE Measurements and Analyses

For modeling the optical constants of PEDOT-rich bulk, we spincast the PEDOT:PSS on both native oxide Si wafer (DOT_nat) and thermally grown oxide (500nm) on Si wafer (DOT_thrm) at 1000 rpm for 1 min. On these films we eliminate the PSS-top layer by sonicating the films in water for 1 min prior to the soft bake step. The optical constants for the PEDOT-rich bulk are developed by fitting a uniaxial B-spline model simultaneously to both DOT_nat and DOT_thrm measurements (**Figure S2A-B**) as accomplished previously on other thin films with tensor optical properties ^35,36^. The resultant optical model from this fit is shown in **Figure S2C**. For the PSS optical model, PSS is mixed with 0.14% of GOPS (to mimic the ratios in PEDOT:PSS solutions) before spincasting on native oxide Si wafer with 800 rpm for 1 min (PSS_nat). An isotropic B-spline model suffices to fit to VASE measurements here to create the optical model (**Figure S2D-E**).

To characterize the bilayer geometry, pristine PEDOT:PSS films are fit (**Fig. 1B**) using the optical models separately developed above for PEDOT-rich bulk and PSS allowing only two open parameters: thickness of these two channels. Thicker films (DOT_thick) are fabricated by more spincasting cycles as described in the methods. All films are spincast on native oxide Si substrate with 2000 rpm for 1 min. The resulting fit for films of all four thicknesses are displayed in **Figure S3.** All fit parameters are shown in **Table S1 and Table S2**.

Alternative models are fit to the thinnest two of the four samples’ “DOT_thick” and “DOT_thin” VASE data to challenge the result of a PSS top layer which are shown in **Figure S4**. **Figure S4A** and **Figure S4C** shows VASE analyses of DOT_thick and DOT_thin samples, respectively, fitted to the same bilayer model except using an isotropic optical model of the PEDOT-rich bulk. The isotropic optical models for the PEDOT-rich bulk are shown in **Figure S4B** and **Figure S4S3D**, respectively. These fits are significantly worse than the uniaxial model despite having significantly more fit parameters. Also, the optical models do not resemble absorbance data. This highlights the necessity to generate uniaxial optical models for conjugated polymer thin films. **Figure S4E** is of the bilayer reversed with the PSS-rich layer below the PEDOT-rich layer and **Figure S4F** is of a single PEDOT-rich layer and no PSS-rich layer. All parameters are shown in **Table S2**. The MSE for both alternative models are significantly higher than that for the primary model where the PSS-rich layer is on top (see **Table S2**). In particular, the reversed bilayer required an unphysical negative thickness of the PEDOT-rich bulk.


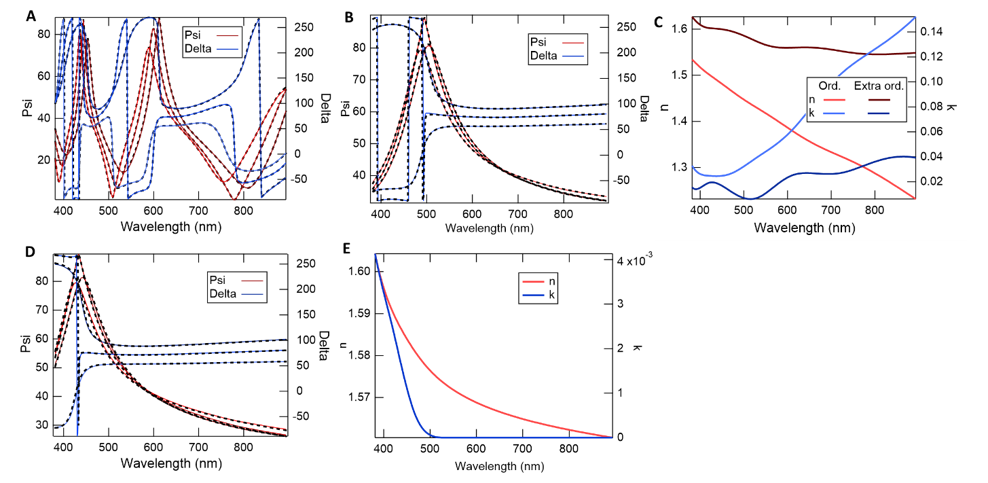


Figure S2: VASE Models of PEDOT Bulk and PSS. VASE measurements of PEDOT-rich bulk (washed via sonication) films on two different Si substrates with: (A) thermally grown oxide and (B) native oxide. (C) The optical constants, n (real) and k (imaginary), from a (dark red and blue) and b (light red and blue). VASE data for (D) PSS with GOPS with optical constants in (E). The PSS solution is prepared by diluting with DI-water in the ratio of 1:10 and mixing 0.14 vol.% of GOPS. All SE measurements are taken at three different angles, 65ᴼ, 70ᴼ, and 75ᴼ to get the average thickness. The solid lines represent the data where the black dotted lines are the model fitting for the data.

**
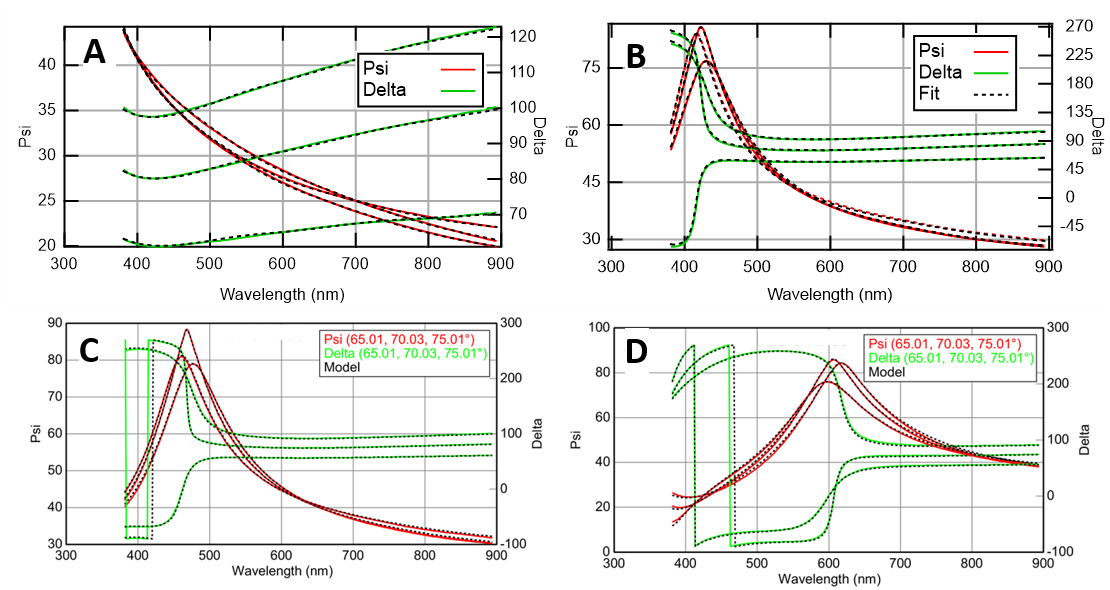
**

Figure S3: VASE Fitting of PEDOT:PSS vs Thickness (A-D) Thinnest to thickest films from varying spincasting conditions. All fits use the same optical models for bulk and interfacial layer with only two parameters (thickness of both layers) open to fit.

Table S1: Parameters from the resulting fits in Figure S3. PSS interfacial layer thickness remains constant between 6-8 nm as the bulk film thickness increases from 48 to 140 nm.

| **Film** | **PSS [nm]** | **Bulk [nm]** |
| --- | --- | --- |
| A | 8.1(2) | 44.0(2) |
| B | 6.1(4) | 82.0(4) |
| C | 6.3(4) | 95.5(4) |
| D | 8.7(4) | 139.3(4) |


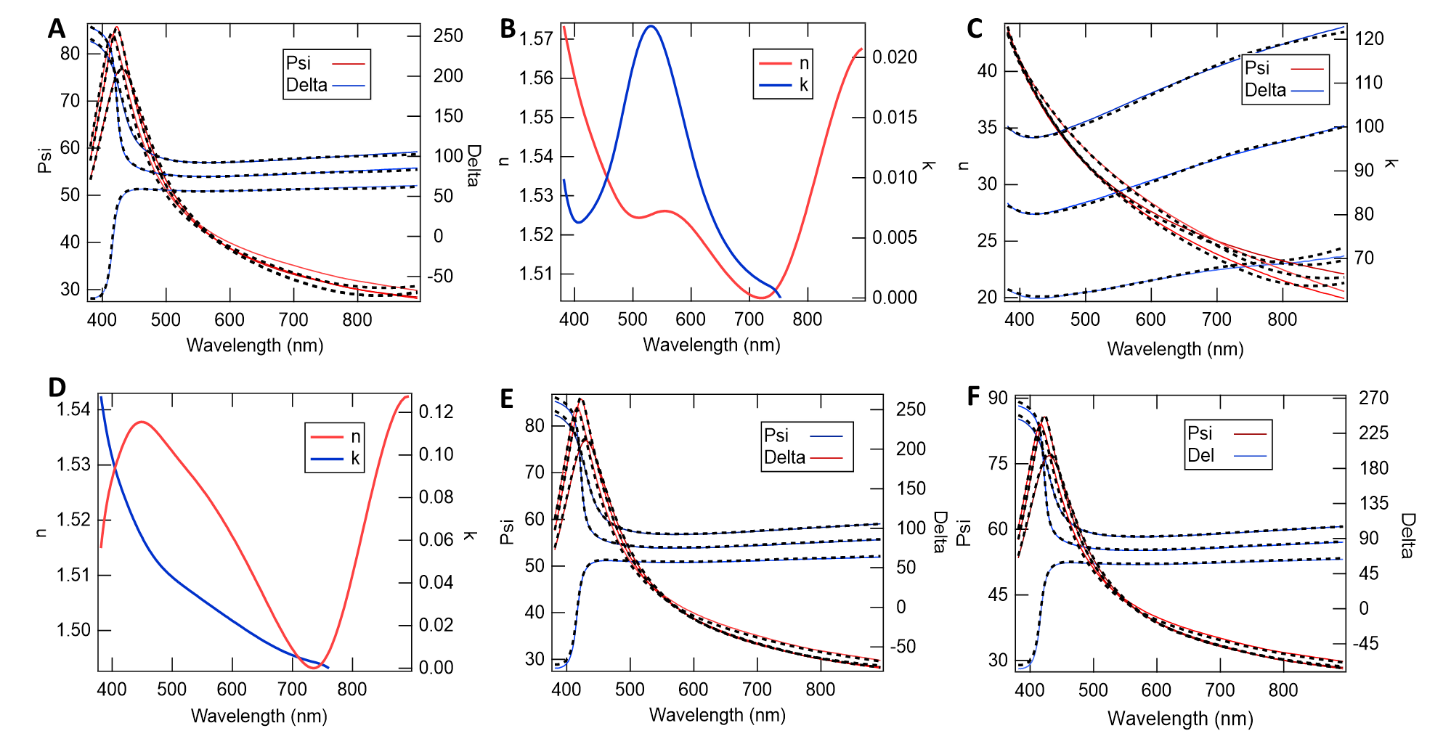


Figure S4: Failed Attempts to fit other potential models. Fitting the as cast PEDOT:PSS film using PSS model atop B-Spline when the solution is spin coated (A) twice and (C) once whose optical constants are in B and D, respectively. Fitting the as cast film (spin coated twice) by (E) reversing the order of model with the PEDOT-rich layer atop a PSS-rich layer and (F) using only PEDOT-rich bulk model. The solid lines represent data and dashed lines are the fitting models.

Table S2: Model fitting parameters of VASE measurements for different samples. (The highlighted fit is described in the main text.)

| Parameters  Substrates | Thickness (nm) | | | E inf | IR Amp | MSE | Angle offset |
| --- | --- | --- | --- | --- | --- | --- | --- |
| DOT_nat (Uniaxial B-spline model) | 111.2 ± 0.2 | | | 1.40 ± 0.03 | 1.92 ± 0.04 | 6.6 | -0.43 ± 0.05 |
| DOT_thrm (Uniaxial B-spline model) | 119.2± 0.2 | | | 1.40 ± 0.12 | 0.39 ± 0.17 |  |  |
| PSS_nat (B-spline) | 81.7 ± 0.4 | | | 1.31 ± 0.03 | 0.03 ± 0.08 | 14.0 | -0.05 ± 0.09 |
| DOT_thick (uniaxial & B-Spline) | PSS | | 6.1 ± 0.4 | N/A | N/A | 12.6 | -0.14 ± 0.020 |
|  | Bulk | | 82.0 ± 0.4 |  |  |  |  |
| DOT_thin (uniaxial & B-Spline) | PSS | | 8.1 ± 0.2 | N/A | 1.92 ± 0.04 | 2.7 | -0.07 ± 0.004 |
|  | Bulk | | 44.0 ± 0.2 |  |  |  |  |
| DOT_thick (Flipping the uniaixal and Bspline model) | Bulk | | -1.0 ± 2.6 | N/A | N/A | 17.5 | -0.14 ± 0.02 |
|  | PSS | | 90.5 ± 2.6 |  |  |  |  |
| DOT_thick (using only uniaxial model) | 89.5 ± 0.02 | | | N/A | N/A | 17.6 | -0.14 ± 0.02 |
| DOT_thin (B-Spline) | PSS | 8.40 ± 2.3 | | N/A | 1.70±0.10 | 6.6 | 0.56 ± 0.100 |
|  | Bulk | 47.8 ± 2.3 | |  |  |  |  |
| DOT_thick (B-Spline) | PSS | 7.5 ± 17.1 | | N/A | 1.60±0.40 | 18.9 | -0.20±0.100 |
|  | Bulk | 76.2±18.5 | |  |  |  |  |

# Conversion from Optical Density to Extinction Coefficient

UV-vis spectroscopy characteristics on PEDOT:PSS film are measured to compare the optical constants from VASE. In UV vis spectroscopy the absorbance is calculated through the Beer-Lambert Law given by

$$\frac{I\left( \lambda\right)}{I_{0}\left( \lambda\right)}=\log\left( -\alpha d \right)$$

Or, $I\left( \lambda\right)=I_{0}\left( \lambda\right) e^{-\alpha d}$

where 𝐼(𝜆) is the intensity measured through the active layer, 𝐼_0_(𝜆) is the measurement of direct beam through a substrate without any active layer, 𝛼 is the film attenuation coefficient, and $d$ is the film thickness. It does not change the physical attributes of the measurement. Further the optical density (OD) is converted to imaginary refractive index $k$.

The OD is defined by the following equation:

$$OD_{10}\left( \lambda\right)=\log_{10} \frac{I_{0}\left( \lambda\right)}{I\left( \lambda\right)}$$

Therefore, combining the last two equations,

$$OD \left( \lambda\right)=\alpha\left( \lambda\right)\frac{d}{\ln10}$$

The absorption coefficient is related to the imaginary refractive index, $\alpha=\frac{4\pi}{\lambda}k$. From this relationship, we get

$$OD \left( \lambda\right) =\frac{4\pi d}{\lambda\ln10}k\left( \lambda\right)$$

$\text{Therefore,} k\left( \lambda\right)=OD\left( \lambda\right)\cdot\lambda\cdot\frac{ln10}{4\pi d}$

The above equation is used to convert the OD from UV vis to k.


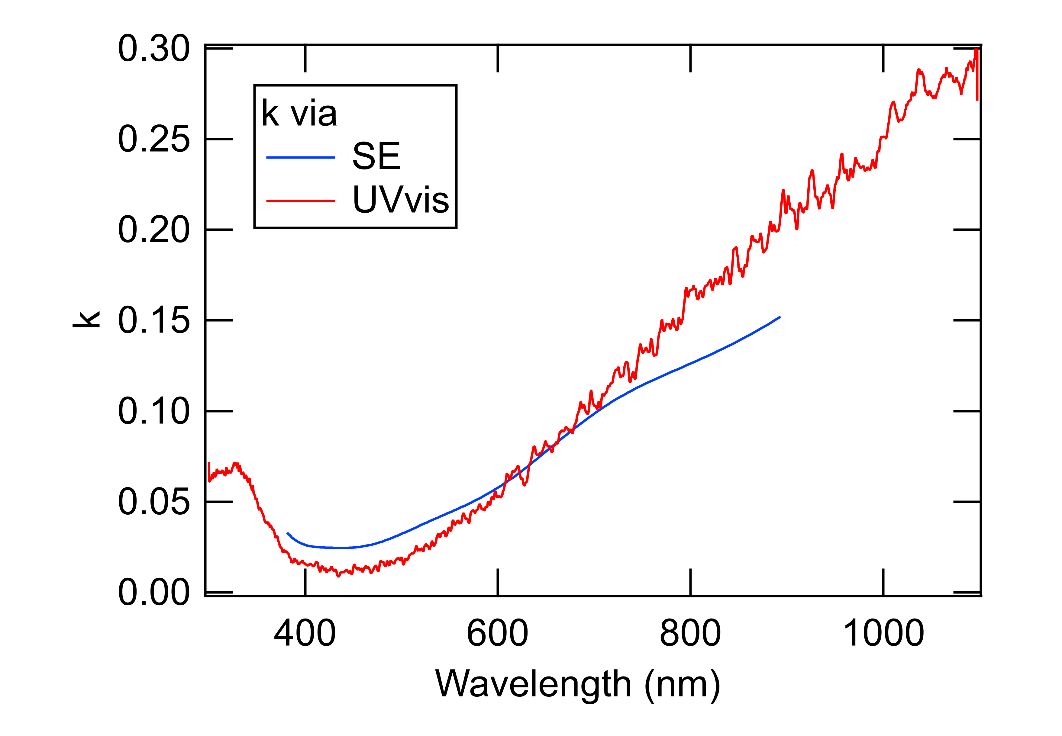


Figure S5: Comparison of Optical Constant (k): The blue trace represents the measurement from VASE measurements and the red is from UV-vis spectroscopy of PEDOT- rich bulk. Identical method as VASE measurements is used to make film for UV-vis spectroscopy measurements.

# Derivation for Ion Mobility


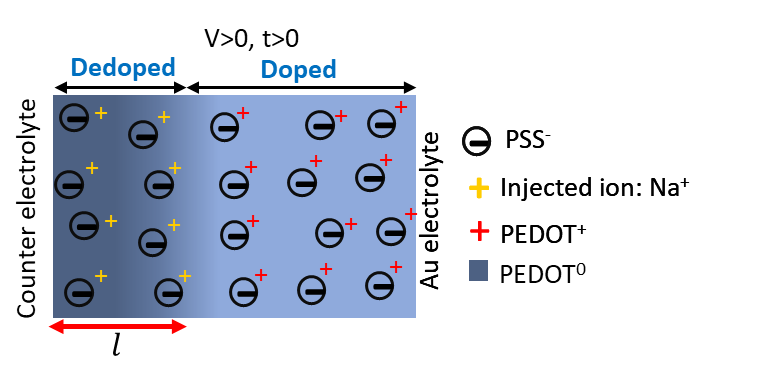


Figure S6: Film Charge and Ion Distribution. Partially (de)doped film when voltage, V is applied. The drift length, $\boldsymbol{l}$ is the distance between the electrolyte/conducting polymer interface to the (de)doped channel segment.

The ion velocity ($v$) can be determined from the ion mobility, $\mu$ times the electric field, $E$:

$v=\mu\cdot E$

Separating and integrating,

$$\frac{dl}{dt}=\mu\cdot\frac{V}{l}$$

$$\int l dl=\int\mu\cdot V dt$$

$l^{2}=2 \mu Vt$ (1)

we derive the relationship between the moving front position $l$ and the ion mobility $\mu$. This comes from the series resistor model developed by the Malliaras Group^17,18^ and shown schematically in **Figure S7**. Here the film is considered as two resistors in a series, where the $R_{l}$ and $R_{c}$ corresponds to the dedoped (blue) and still doped (clear) segments. A few assumptions are made in this model: The ion mobility is lower than the hole mobility and there is enough electrolyte to neglect ion depletion.


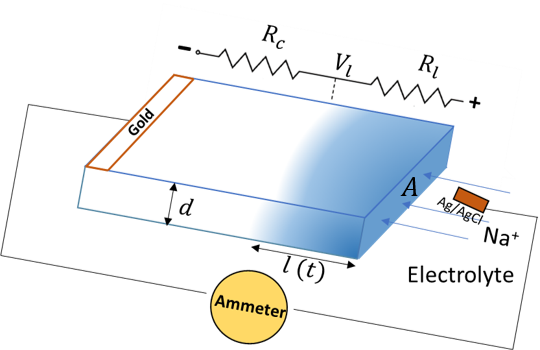


Figure S7: Series Resistor Model and DC Current Setup. Schematic of a dedoped film while voltage is applying with equivalent circuit diagram.

**Modifying the Series Resistor Model to include the PSS Superhighway.**

In the case of the PSS top layer “superhighway” in parallel with ion current in the bulk PEDOT-rich layer below, the series resistor model is modified as shown in **Figure S8**. $R_{l}$ now becomes the equivalent parallel resistance of the interface “$i$" and bulk “$b$” layers and only an effective mobility $\mu_{eff}$ is extracted from the ECMF experiment which, like resistance, is dominated by the interfacial superhighway if the ion mobility of the top layer is significantly higher than that of the bulk. $\mu_{eff}$ can be calculated by starting with calculating the parallel resistance:


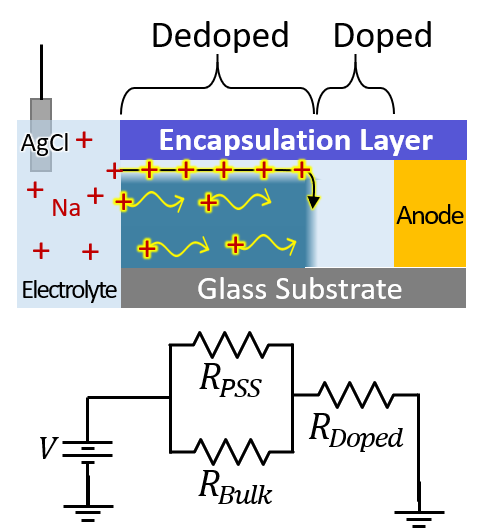


Figure S8: Expanded model to include effects of the interfacial PSS channel. TOP: Crossectional schematic of the two parallel channels. BOTTOM: Equivalent circuit of the parallel channels.

$$\frac{1}{R_{l}}=\frac{1}{R_{i}}+\frac{1}{R_{b}}=\frac{A_{i}\rho_{i}\mu_{i}}{l}+\frac{A_{b}\rho_{b}\mu_{b}}{l}=\frac{\Sigma\rho A\mu}{l}$$

where $\rho=ep$ is the charge density and $\Sigma$ means the sum over the two resistors’ parameters.

Ion velocity is related to the current by $v=I/\rho A$. Using Ohm’s Law $I=V/R$, this equation can be altered for the case of parallel resistors, and then $R$ can be replaced with the equivalent resistance calculated above:

$$v=\frac{V}{R_{l}}\frac{1}{\Sigma\rho A}=\frac{V \Sigma\rho A\mu}{l \Sigma\rho A}=E\cdot\mu_{eff}$$

|  | $\mu_{eff}=\frac{\Sigma\rho A\mu}{\Sigma\rho A}$ | (2) |
| --- | --- | --- |

This yields that the effective mobility $\mu_{eff}$ is the average of the mobilities in the two parallel channels weighted by their crossectional area $A$ and charge density $\rho$.

Experimentally, one can increase the thickness of the bulk, increasing $A$ while holding $\rho$ constant. This will cause $\mu_{eff}$ to skew increasingly toward the intrinsic mobility of the bulk $\mu_{eff}\to\mu_{b}$. This is indeed what is seen when the bulk layer is made systematically thicker as shown in ***Figure S9***.

**
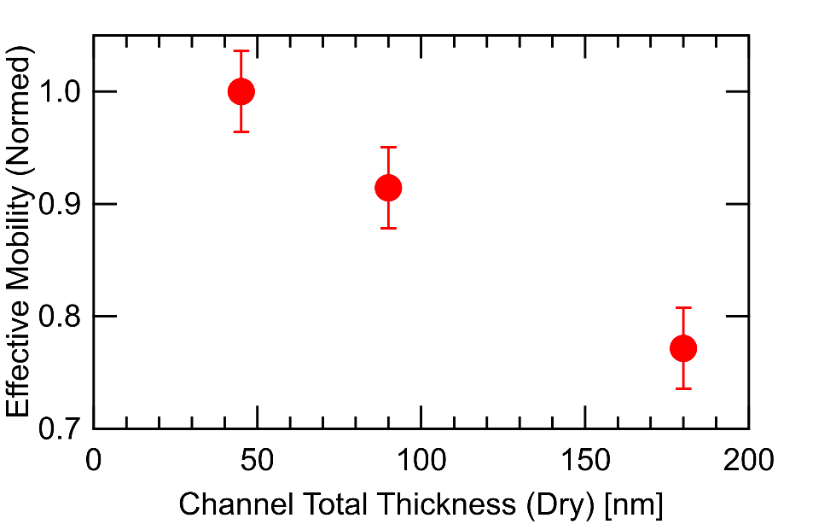
**

Figure S9: Moving Front Velocity Slows with PEDOT Thickness, resulting in a lower effective mobility with thickness. Thicker films are attained from multiple castings of the PEDOT:PSS solution with a subsequent soft bake. Notably, previously published mobilities^12,17^ report 400 nm thick channels, while ours are measured at 80-90nm dry.

# Conductivity Measurements

We have measured the ion conductivity $\sigma_{ion}=e\mu p$ of the dedoped film simultaneous to the optical ECMF measurement. **Figure S7** depicts the experimental setup that measures current through the counter electrode simultaneous to the optical ECMF measurement. To calculate $\sigma_{ion}$ from mobility $\mu$ we need to also measure the ion density $p$. We can do this by measuring the current through the device. Ohm’s law reveals $\sigma_{ion}$ through the measured current, segment length $l$, and crossectional area $A$:

$$I=\frac{V\sigma A}{l}$$

Replacing$l$ from equation (1) and combining with the definition of conductivity above results in a function of ion density:

$I= epA\sqrt{\frac{V\mu}{2t}}$ (3)

We can solve this for the ion density:

$$p=\frac{I}{eA}\sqrt{\frac{2t}{V\mu}}$$

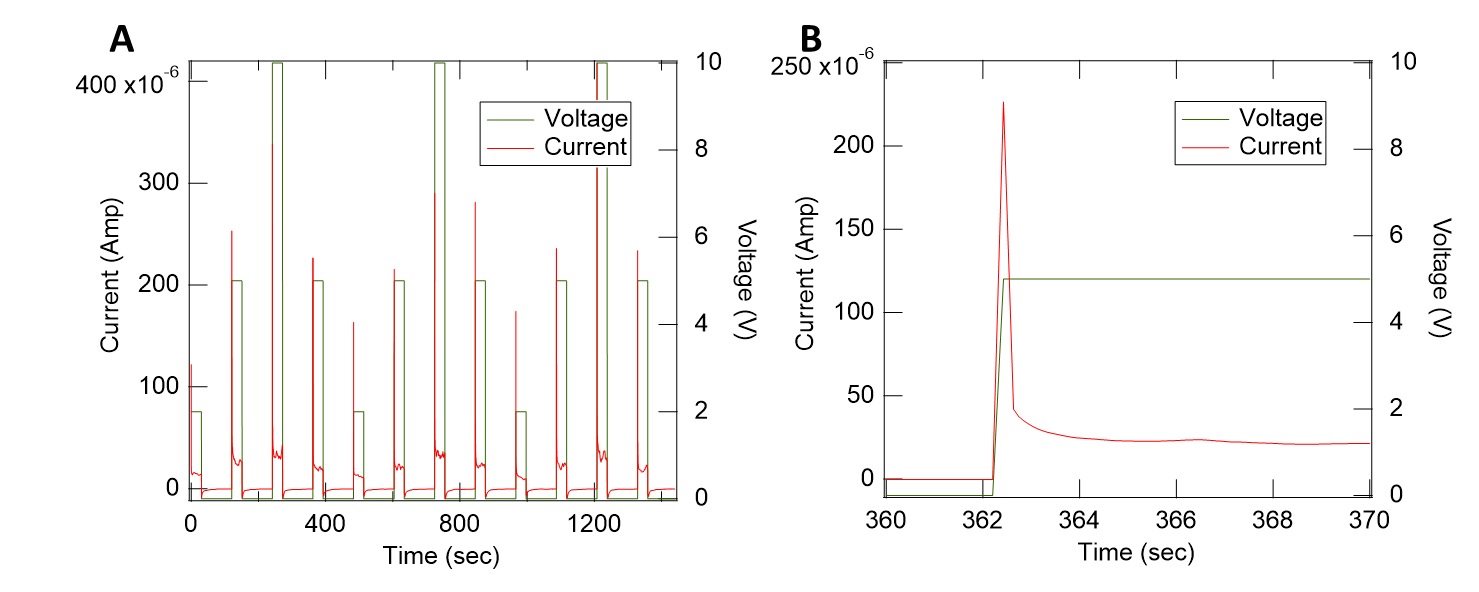


Figure S10: DC Current Measurement (A) Current vs time graph for twelve dedoping/redoping cycles and (B) zoomed in version of a dedoping cycle from electrical measurements of a mixed conduction device with PVA encapsulation layer. The green and red traces represent the voltage and current, respectively.

**Fig. S7** depicts the automated ECMF experiment. **Fig. S7a** current vs time graph of 12 cycles of dedoping/redoping where the dedoping is hold for half a minute and the redoping was hold for four times longer to return all the ions back to the electrolyte. The current spikes up in the beginning of time for each cycle of doping which can be seen clearly in **Fig. S7b** which is a zoomed to show beginning few seconds of dedoping cycle while 5V is applied across the device. According to the two-resistor model, at $t=0$, the dominant resistor is $R_{c}$ because there is no $R_{l}$ yet (no ion injection). Therefore, at that instant of time the current is dominated by the hole transport. After that, as ions enters into the film, $R_{l}$gets bigger and eventually becomes the dominant resistance. Therefore, the current declines back down in nominal level. Afterward, during the removal of bias the current spikes down again while redoing and shows negative value which eventually comes back to zero current (**Fig. S7a**).

# Solubility analysis of the barrier materials

Table S3 δ for different coating materials and water: ^37–40^

| Material | δ_d_  (MPa^1/2^) | δ_p_  (MPa^1/2^) | δ_h_  (MPa^1/2^) | R_0_  (MPa^1/2^) | RED |
| --- | --- | --- | --- | --- | --- |
| PVC | 20.90 | 11.30 | 9.60 | 13.70 | 2.25 |
| PMMA | 17.70 | 6.70 | 6.20 | 8.59 | 3.02 |
| SU8 | 18.10 | 11.40 | 9.00 | 9.10 | 3.27 |
| PVA | 18.20 | 7.50 | 8.30 | 3.50 | 7.70 |
| Water | 6.00 | 15.30 | 16.70 |  |  |

A solubility analysis for measuring the hydrophobicity of the encapsulating layer is obtained from relative energy density (RED) using Hansen solubility parameters (HSPs) which are the extension of the Hildebrand solubility parameter, δ. δ defines as the square root of the ratio of cohesive energy to the molar volume of the liquid. δ is well characterized by three cohesive components: dispersion force component (δ_d_), polar force component (δ_p_), and hydrogen bonding component (δ_h_). These three Hansen parameters are needed for a certain material to define the cohesive energy density. By considering each Hansen parameter as one of the three coordinates, a 3D spherical area of solubility is fabricated where the radius is called the interaction radius (R_0_). The value of δ_d_, δ_p_, and δ_h_ for different components are given in table S3. The distance between two substances 1 and 2, is defined as R_a_:

R_a_^2^ = 4 (δ_d1_ - δ_d2_)^2^ + (δ_h1_ – δ_h2_)^2^ + (δ_p1_ – δ_p2_)^2^

The smaller the value of R_a_, the solvents are more likely to dissolve into each other. The preferred quantity to define the solubility is termed relative energy density (RED= R_a_ /R_0_). As the RED value increases the hydrophobicity also increases. Therefore, using the values from SI Table 3, the calculated RED values show that the hydrophobicity of coating materials increases as PVA<SU8<PMMA<PVC.

# Mobility Measurements for Different Ions

**
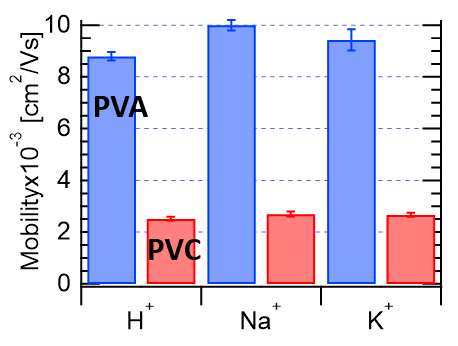
**

Figure S11: Encapsulation Gating Effect for Three Ions. The hydrophobic (PVC) and hydrophilic (PVA) gating effect is seen H^+^, Na^+^, and K^+^ alike using electrolytes H-PSS, NaCl, mand KCl, respectively.

# Identifying Encapsulation Layer Dewetting

**
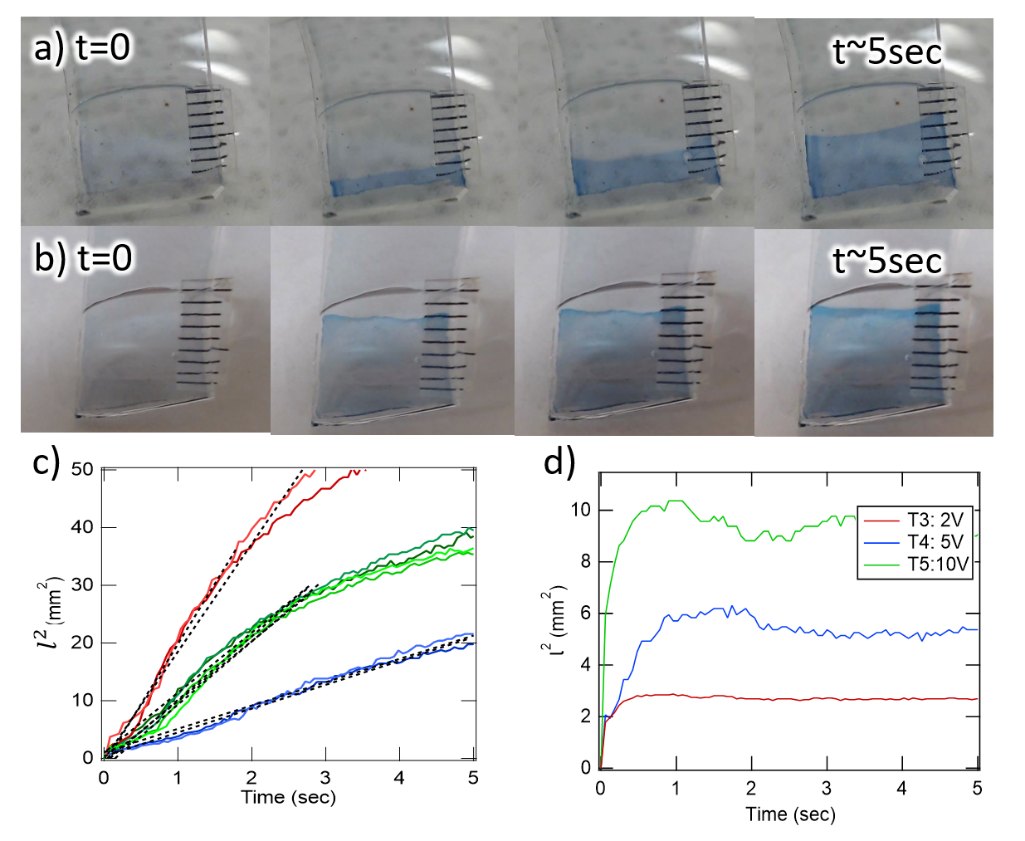
**

Figure S12: Comparison between video frame and transients of Normal and Dewetted Encapsulation Layers. (a & b) Four video frames in the first 5 seconds of the ECMF experiment. In the normal (non-dewetted) device (a), the blue region expands due to a well-defined front that moves monotonically upward. In the device with a dewetted encapsulation layer (b), the dedoped region fades in over time with a non-moving front due to transport of ions only downward into the channel. (c & d) depict the moving front position ($\boldsymbol{l}$) transients during the experiment for a normal device (c) and a device where the encapsulation layer is dewetted (d). Again for dewetted films, the maximum extent of dedoping appears nearly instantaneously, and the slope is not proportional to voltage. These two situations are always clearly distinguishable, and data from the dewetted ECMF experiments are always thrown out.

# Swelling Experiments

The high mobility of Na ions in PEDOT:PSS demands investigation for possible mechanism. Dedoping the film makes it hydrated which causes the swelling of the film. However, too much swelling could result in dewetting of the film meaning the encapsulation loses contact with the film. Therefore, the water as well as the ions from the electrolyte rush into the film as soon as the voltage is applied and causing mobility to be very high.

Previously published report showed that an uncrosslinked (without GOPS), unwashed film swells around 155% after wetting and a crosslinked film via 1% GOPS swells around 35% ^17^. All our PEDOT:PSS films are made with 0.14% GOPS. Now, to measure the thickness of the PEDOT:PSS, devices with and without water rinsing using a PVA (most hydrophilic) and PVC (most hydrophobic) encapsulation layers are made. VASE measurements are taken on these devices before and after dedoping with NaCl electrolyte via ECMF experiment.

Because of the presence of the water in the film we cannot use the optical constants of PEDOT-bulk and pure PSS for measuring the thickness of the swelled channel. Therefore, we first model the optical constants for PVA and PVC. To do that we spincast PVA and PVC on native oxide Si wafer and the VASE measurements are fitted to a B-Spline.


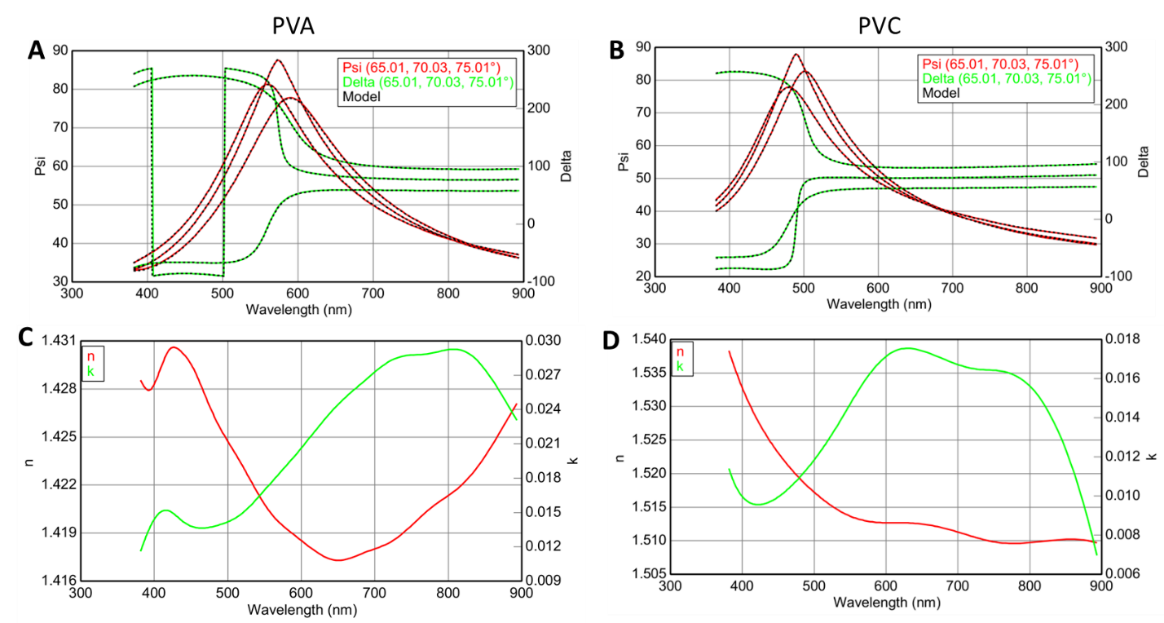


Figure S13: Optical Constants of Encapsulation. VASE Measurement of (A) PVA and (B) PVC with optical constant in C and D, respectively. The thickness of PVA and PVC is 134.07nm (MSE= 2.92) and 101.22nm (MES= 1.92), respectively.

To measure the thickness of the swelled film a uniaxial B-Spline model is used with the optical constant of PVA or PVC atop. The thickness of the encapsulation layer and the channel are open to vary. Some of the example graphs are shown below with the optical constants from the PVA coated device:


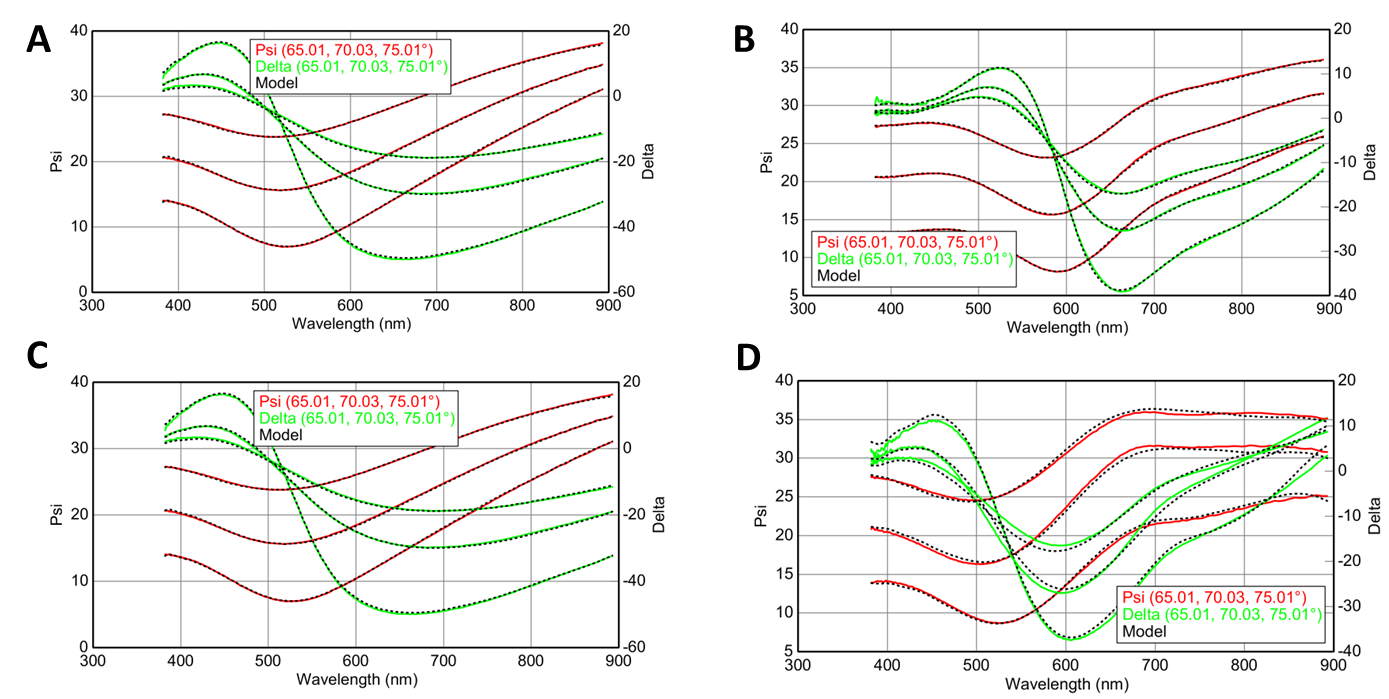


Figure S14: Thickness Measurements of Swelled Film. The VASE measurements of the film on the PVC coated devices when (A) dry and (B) swelled with PSS, (C) dry and (D) wet without PSS present in the device on glass substrate. All fitting parameters are shown in Table S3.

Table S4: Parameters from VASE for swelling experiments

| Sample Condition | Parameter  Dedoping  Trial number | Thickness of encapsulation (nm) | Channel thickness (nm) | MSE |
| --- | --- | --- | --- | --- |
| With PSS | 0^th^ | 134.68 | 89.88 | 2.84 |
|  | 7^th^ | 133.41 | 166.30 | 2.12 |
| W/o PSS | 0^th^ | 132.77 | 79.87 | 2.73 |
|  | 7^th^ | 134.52 | 146.11 | 10.37 |

Table S 5: Thickness of swelled bulk and interfacial channel

| Encapsulation Layer | Sample Condition | Dedoping trial number | Thickness of channel, $t$ (nm) | Bulk swelling, $S_{bulk}$ (%) | PSS swelling, $S_{PSS}$ (%) |
| --- | --- | --- | --- | --- | --- |
| PVA | With PSS | 0^th^ | 89.88 | 84.82 | 101.70 |
|  |  | 7^th^ | 166.30 |  |  |
|  | W/oPSS | 0^th^ | 79.87 | 82.93 |  |
|  |  | 7^th^ | 146.11 |  |  |
| PVC | With PSS | 0^th^ | 86.09 | 81.75 | 83.07 |
|  |  | 7^th^ | 156.47 |  |  |
|  | W/o PSS | 0^th^ | 81.60 | 81.68 |  |
|  |  | 7^th^ | 148.25 |  |  |

Here, $S_{bulk}=$ $\frac{t^{7th}-t^{0th}}{t^{0th}}X 100\%$ and $S_{PSS}= \frac{\left( t_{PSS}^{7th}-t_{\frac{w}{oPSS}}^{7th} \right)-\left( t_{PSS}^{0th}-t_{\frac{w}{oPSS}}^{0th} \right)}{t_{PSS}^{0th}-t_{\frac{w}{oPSS}}^{0th}}X100\%$

# Comparing the Density of Anion (S0_3_^-^) to Na^+^

We have calculated the number density of sulfonate anions in pure dry PSS, hydrated/swollen PSS, PSS in bulk PEDOT:PSS, and PEDOT:PSS crystal to see how resonable is the Na ion density ($p$) we calculated in **Fig. 1H**.

Molecular weight of a PSS monomer, $M=206.2 \frac{g}{mol}$

Mass density of PSS, $\rho=0.8019\frac{g}{cm^{3}} ADDIN ZOTERO\_ITEM CSL\_CITATION \{"citationID":"kBgGsREa","properties":\{"formattedCitation":"\backslash\backslash super 41\backslash\backslash nosupersub\{\}","plainCitation":"41","noteIndex":0\},"citationItems":[\{"id":"t9CFLuGE/28Uog4QC","uris":["http://zotero.org/users/7436350/items/BHRIUJCD"],"itemData":\{"id":873,"type":"webpage","abstract":"Poly(sodium 4-styrenesulfonate) (PSS) | Poly (sodium 4-styrenesulfonate) (PSS) a cation exchange polyelectrolyte | Reduction of graphite oxide in the presence of PSS has been reported for the synthesis of polymer-coated graphitic nanoplatelets | Buy chemicals and reagents online from Sigma Aldrich","language":"en","title":"Poly(sodium 4-styrenesulfonate) average Mw 70,000, powder 25704-18-1","URL":"http://www.sigmaaldrich.com/","accessed":\{"date-parts":[["2022",8,18]]\}\}\}],"schema":"https://github.com/citation-style-language/schema/raw/master/csl-citation.json"\}$^41^

Number density of dry PSS:

$$n_{PSS}=\frac{\rho}{M}=0.8019\left[ \frac{g}{cm^{3}} \right]\cdot\frac{1}{206.2}\left[ \frac{mol}{g} \right]\cdot6.023X{10}^{23}\left[ \frac{monomer}{mol} \right]$$

Therefore,

$$n_{PSS}=2.34\times\frac{{10}^{21} monomers}{cm^{-3}}$$

Swelling causes $n_{PSS}$ to reduce. We have measured ~102% swelling. Therefore,

  $n_{PSS}^{Swell}=1.16\times\frac{{10}^{21}}{cm^{3}}$

In PEDOT:PSS average blend (1:2.5 w/w) we have measured 83% swelling resulting in

$n_{PSS}^{Blend}=\frac{2.5}{3.5}\cdot\frac{100}{183} n_{PSS}=9.1\times\frac{{10}^{20}}{cm^{3}}$

In a (dry) PEDOT:PSS crystal there is reported 1 PSS to every 4 PEDOT ^17,42^. Therefore,

$$n_{PSS}^{xtl}=\frac{n_{PSS}}{5}=4.7\times\frac{{10}^{20}}{cm^{3}}$$

# Reproducible Effect of UV Ozone on PMMA Devices


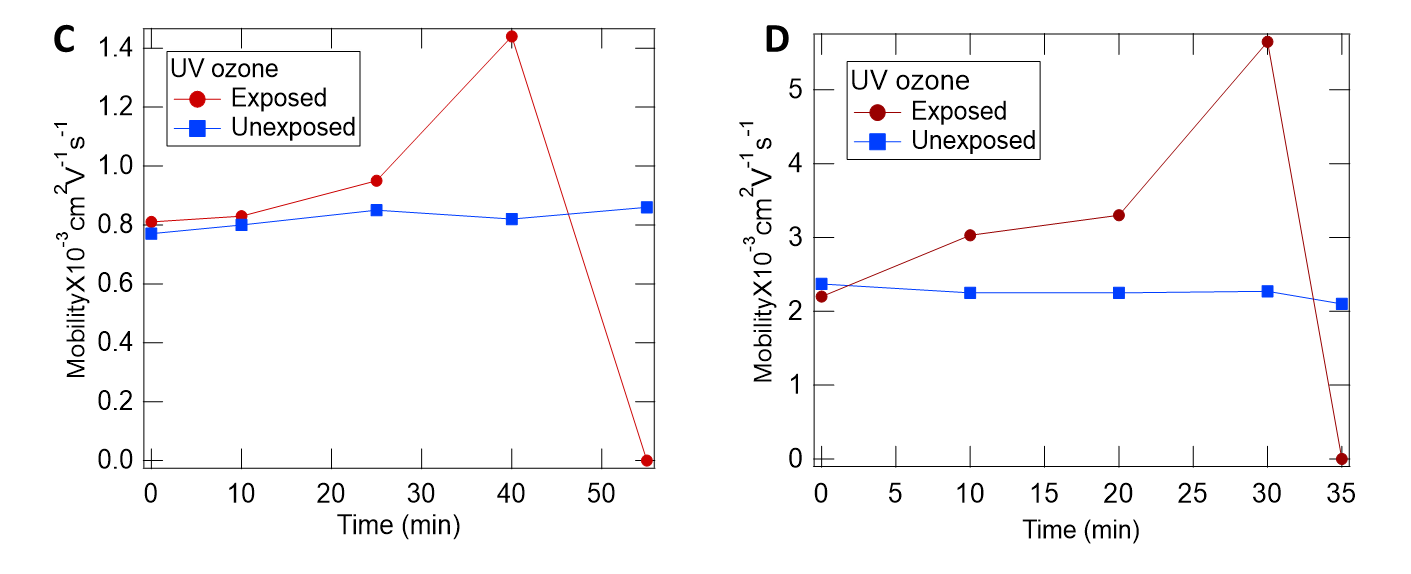


Figure S15: Reproducible effect of UV Exposure on PMMA Devices. WCA of PMMA when the device is (A) exposed via UV ozone and (B) unexposed up to 35 mins. Both WCA and MFE are done on the same devices. Mobility measurement on two separate devices showing in C and D. This shows the same trend as in Fig. 3B.

# Electrical Measurement of Mixed Conduction Devices


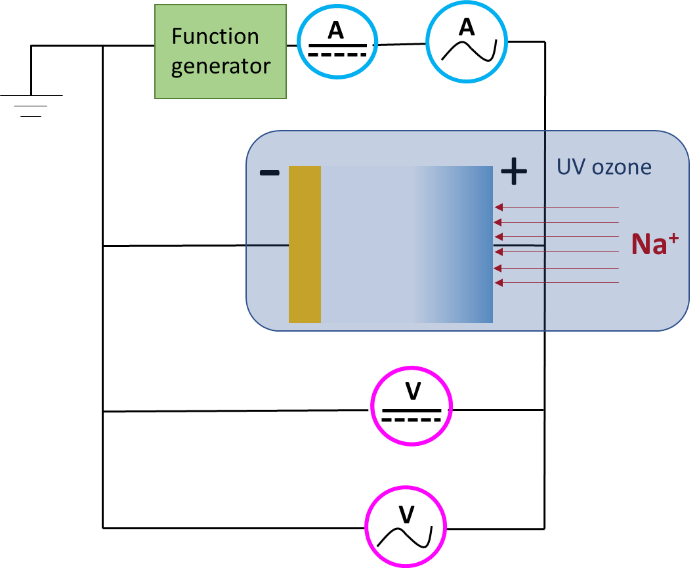


Figure S16: Experimental Setup for AC Current Measurements. Schematic of setup for AC current measurement over time while dedoping the device with 5V with UV ozone. A function generator is used to provide the DC voltage. The four multi meters show the measured electrical signal.

Taking the derivative of Equation 3 reveals how ion mobility affects an AC current.

$$\frac{dI}{dt}= \frac{epA\sqrt{V}}{\sqrt{8t^{3}}} \sqrt{\mu}$$

The above equation shows that AC current is proportional to the square root of ion mobility. We, therefore, use the peak in AC current to sense when high ion mobility is activated by the oxidative chemical reaction in buried channel of our devices. Data with various PMMA thicknesses are shown in Figure S17 below depicting the linear relationship between the reaction time and layer thickness.

Figure S17: AC Current transients for devices with different PMMA Gate thicknesses. Thicknesses measured via VASE. Peaks in the current transients depict when the oxidative reaction has traveled through the PMMA gate layer and thus senses the reaction time.

# Solvent testing for orthogonality to PSS

It is important that the solvents we use to dissolve the encapsulation materials do not affect the PSS interfacial layer in the film. To see if these solvents affect the PSS interfacial layer, VASE measurements are performed on the PEDOT:PSS sample with and without the solvents atop. A PEDOT:PSS film is made (same procedure as described in methods) on native oxide Si substate with dimension of ~3”X3”. First a measurement via VASE is taken to determine the thickness of the PEDOT and PSS interfacial channel (**Fig. S13A** and **Table S5**). Then this film is cleaved into three parts to make three samples. Each of the sample is spin coated with three of the solvents i.e., chloroform, toluene, and THF, with 500 rpm for 1 min on top of PEDOT:PSS film. Cutting three samples from one gives more control over the thickness and reduces the uncertainty. Afterward, the thickness of the PEDOT and PSS interfacial layer are measured from three of the samples (**Fig. S13B-D**). The resulting VASE data shown in **Table S5** represents the thickness of PSS interfacial channel remains essentially identical with and without the solvent Chloroform and Toluene which are used to dissolve PVA and PMMA, respectively. Therefore, these two solvents do not affect the PSS interfacial channel and several nm of PSS interfacial is available to transport the ion through them. However, solvent for PVC, THF, reduces the PSS thickness to less than half a nm which could mean that PSS interfacial channel is dissolved in PVC. Therefore, no PSS interfacial channel is available for ions to move. To solve this problem, another solvent, Cyclohexanone, is used. The thickness of PSS interfacial channel without and with Cyclohexanone is again measured (**Fig. S13E-F**) which confirms that the presence of PSS channel is present on the top of the PEDOT:PSS film.


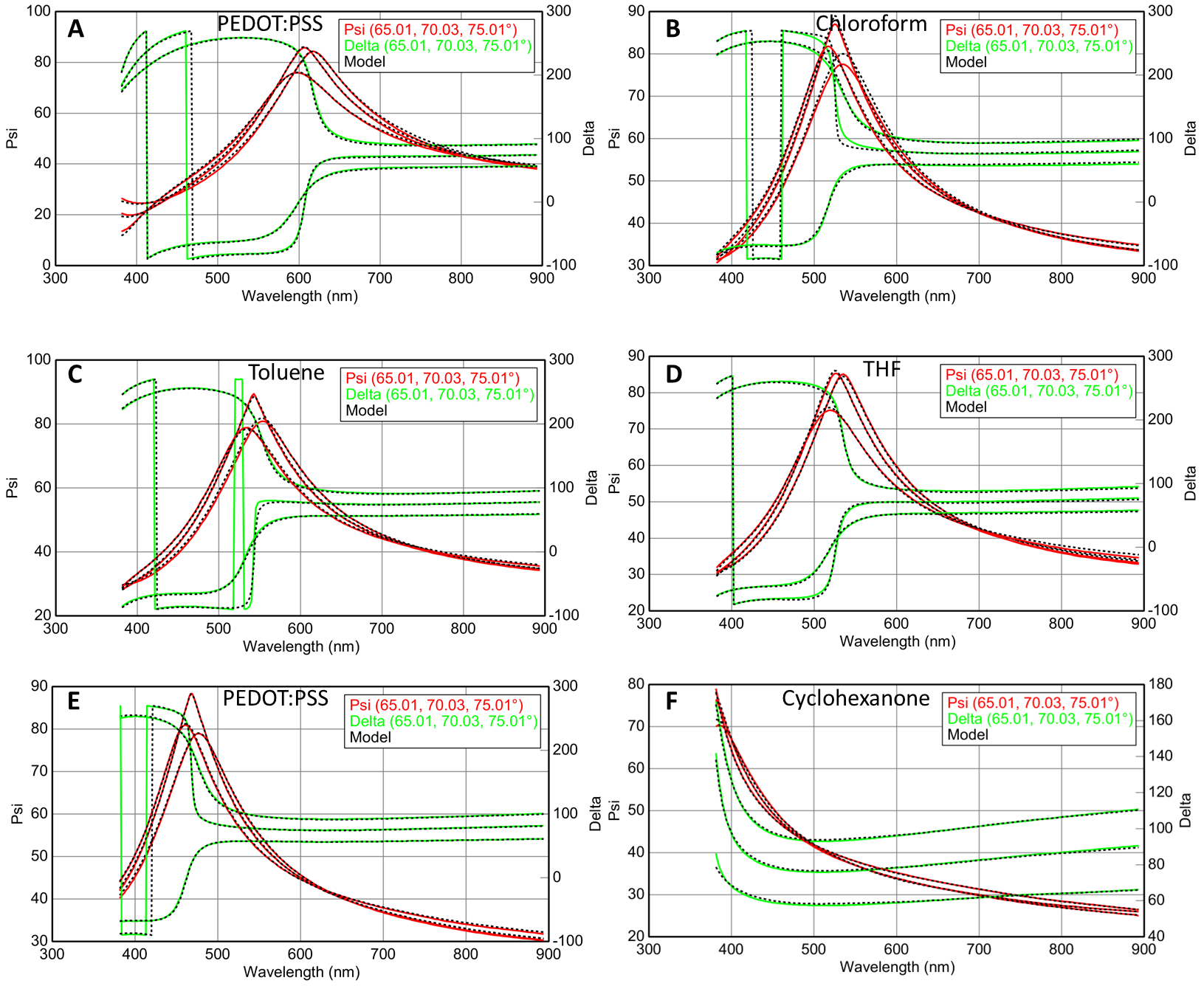


Figure S18: PSS Thickness Measurement with Solvents. Thickness measurement of A) pure PEDOT:PSS, PEDOT:PSS coated with B) Chloroform, C) Toluene, D) THF. Thickness measurement of E) pure PEDOT:PSS film and F) coated with Cyclohexanone.

Table S6: Thickness of PEDOT:PSS Film with and w/out Solvent Using VASE


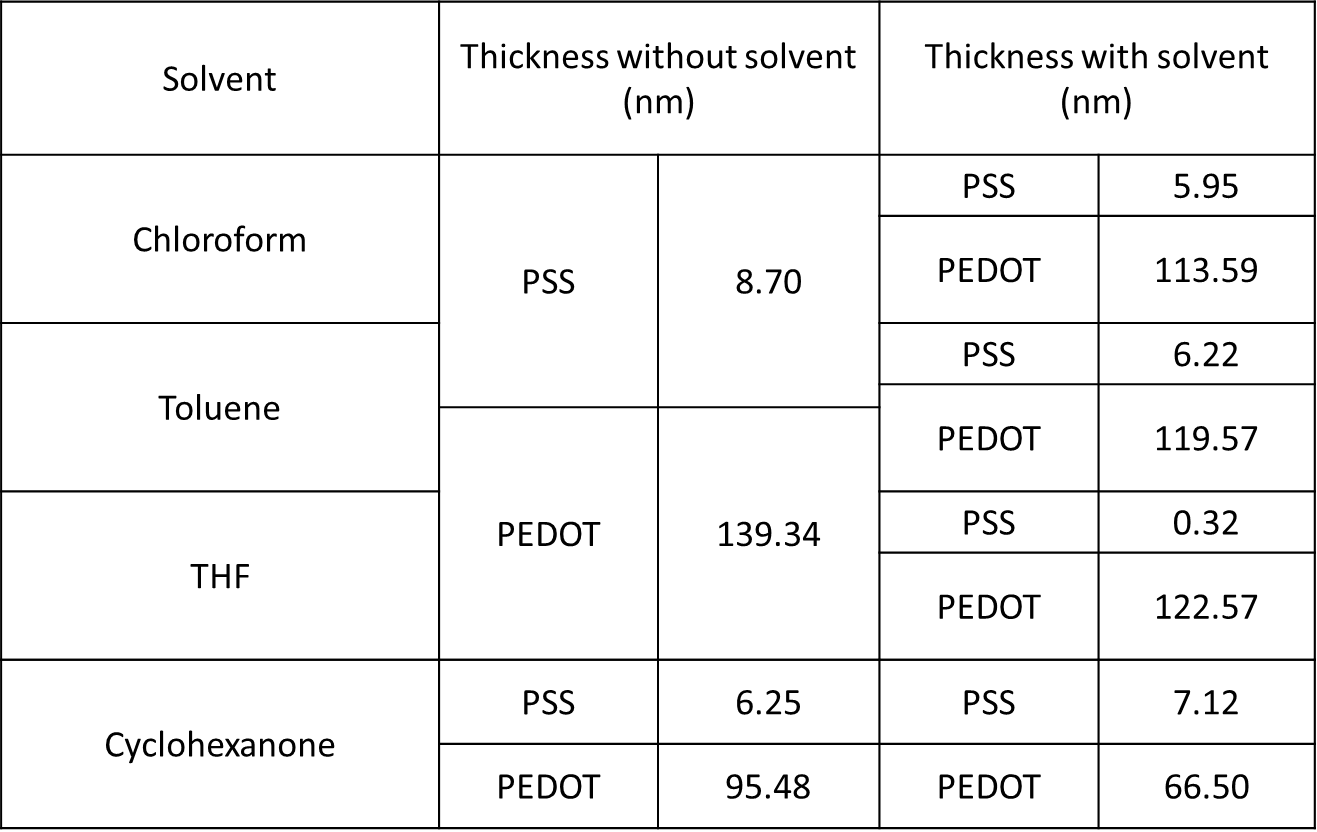


**Supporting Information References**

35. Tammer, M. & Monkman, A. p. Measurement of the Anisotropic Refractive Indices of Spin Cast Thin Poly(2-methoxy-5-(2′-ethyl-hexyloxy)-p-phenylenevinylene) (MEH–PPV) Films. *Adv. Mater.* **14**, 210–212 (2002).

36. Campoy-Quiles, M., Alonso, M. I., Bradley, D. D. C. & Richter, L. J. Advanced Ellipsometric Characterization of Conjugated Polymer Films. *Adv. Funct. Mater.* **24**, 2116–2134 (2014).

37. Salman, R. K. & Salih, J. M. A New Approach to Estimate Hansen Solubility Parameters Using Maple Software. *Int. J. Mater. Sci. Appl.* **5**, 183 (2016).

38. Hansen, C. M. *Hansen Solubility Parameters: A User’s Handbook, Second Edition*. (CRC Press, Boca Raton, 2007). doi:10.1201/9781420006834.

39. Kang, D.-Y., Kim, C., Park, G. & Moon, J. H. Liquid immersion thermal crosslinking of 3D polymer nanopatterns for direct carbonisation with high structural integrity. *Sci. Rep.* **5**, 18185 (2015).

40. Burke, J. Solubility Parameters: Theory and Application. https://cool.culturalheritage.org/coolaic/sg/bpg/annual/v03/bp03-04.html (1984). Accessed: October 2022.

41. Poly(sodium 4-styrenesulfonate) average Mw 70,000, powder 25704-18-1. <http://www.sigmaaldrich.com/>. Accessed October 2022.

42. Andreas Elschner, Stephan Kirchmeyer, Wilfried Lovenich, Udo Merker, & Knud Reuter. PEDOT: Principles and Applications of an Intrinsically Conductive Polymer. *Routledge & CRC Press* (2011).
